# Health-related quality of life in patients with aggressive non-Hodgkin lymphoma: results from the PETAL trial

**DOI:** 10.1007/s00277-025-06402-1

**Published:** 2025-05-21

**Authors:** Ulrich Dührsen, Gabriele Prange-Krex, Regina Moeller, Harald Held, Gerhard Heil, Andreas Schwarzer, Stefan Mahlmann, Ariane Dienst, Matthias Sandmann, Georg Maschmeyer, Jochen Schütte, Dennis Hahn, Michael Heike, Michael Nonnemacher, Christine Hanoun, Andreas Hüttmann, the PETAL trial investigators

**Affiliations:** 1 Klinik für Hämatologie und Stammzelltransplantation, Universitätsklinikum Essen, Essen, Germany; 2 Onkologische Gemeinschaftspraxis, Dresden, Germany; 3 Hämatologisch-onkologische Gemeinschaftspraxis, Halle, Germany; 4 Klinik für Hämatologie/Onkologie und Nephrologie, Friedrich-Ebert-Krankenhaus, Neumünster, Germany; 5 Klinik für Hämatologie und Onkologie, Klinikum Lüdenscheid, Lüdenscheid, Germany; 6 Onkopraxis Probstheida, Leipzig, Germany; 7 Klinik für Innere Medizin 1, Westpfalz-Klinikum, Kaiserslautern, Germany; 8 Klinik für Hämatologie, Onkologie und Klinische Immunologie, Universitätsklinikum Düsseldorf, Düsseldorf, Germany; 9 Klinik für Innere Medizin III, Petrus-Krankenhaus, Wuppertal, Germany; 10 Klinik für Hämatologie, Onkologie und Palliativmedizin, Klinikum Ernst von Bergmann, Potsdam, Germany; 11 Klinik für Onkologie, Hämatologie und Palliativmedizin, Marienhospital, Düsseldorf, Germany; 12 Klinik für Hämatologie, Onkologie, Stammzelltransplantation und Palliativmedizin, Klinikum Stuttgart, Stuttgart, Germany; 13 Medizinische Klinik Mitte, Klinikum Dortmund, Dortmund, Germany; 14 Institut für Medizinische Informatik, Biometrie und Epidemiologie, Universität Duisburg-Essen, Essen, Germany

**Keywords:** Aggressive non-Hodgkin lymphoma, Chemotherapy, Diffuse large B-cell lymphoma, Positron emission tomography, Quality of life

## Abstract

When different therapies provide similar cure rates, health-related quality of life (HRQoL) may become crucial for the choice of treatment. In the Positron Emission Tomography-guided Therapy of Aggressive non-Hodgkin Lymphomas (PETAL) trial, we compared six cycles of R-CHOP with or without two extra doses of rituximab in prognostically favorable interim PET (iPET)-negative patients, while eight cycles of R-CHOP were compared with two R-CHOP cycles followed by six cycles of a more intensive protocol in prognostically unfavorable iPET-positive patients. As reported previously, treatment intensification did not improve outcome. HRQoL was assessed using the EORTC QLQ-C30 questionnaire. Pretreatment questionnaires were obtained from 558 out of the 862 participants (64.7%). Pretreatment HRQoL was significantly worse than in the general population. It was associated with age, gender, B symptoms, International Prognostic Index (IPI) and total metabolic tumor volume (TMTV). Physical and cognitive functioning predicted survival independent of IPI or TMTV. During treatment, some domains remained stable (e.g., cognitive functioning, nausea/vomiting), while others improved (e.g., emotional functioning, pain) or deteriorated (e.g., physical functioning, role functioning, fatigue). At the end of treatment, HRQoL was better in patients with controlled disease than in patients with progressive disease and better for iPET-negative patients than for iPET-positive patients. During follow-up, all HRQoL domains returned to levels similar to those reported for the general population. Differences between randomized treatment arms were not observed. The longitudinal data need to be interpreted with caution, because decreasing participation resulted in a selection of patients with increasingly good outcomes. ClinicalTrials.gov no. NCT00554164 (registered 11/5/2007).

**Supplementary Information:**

The online version contains supplementary material available at 10.1007/s00277-025-06402-1.

## Introduction

The goal of treatment for aggressive non-Hodgkin lymphoma is cure. When different therapies provide similar cure rates, health-related quality of life (HRQoL) may become crucial for the choice of treatment. Trials comparing therapies should therefore include a HRQoL assessment [1–4].

In the Positron Emission Tomography-guided Therapy of Aggressive non-Hodgkin Lymphomas (PETAL) trial, we divided the study population into groups according to the interim PET (iPET) response. In the prognostically favorable iPET-negative group, we compared six cycles of R-CHOP (rituximab, cyclophosphamide, doxorubicin, vincristine, prednisone) alone with six R-CHOP cycles followed by two doses of rituximab. In the unfavorable iPET-positive group, patients remained on R-CHOP for a total of eight cycles or switched from R-CHOP to a more intensive protocol. None of the treatment changes improved outcome [5, 6].

In the PETAL trial, HRQoL was assessed at pre-defined times between diagnosis and 12 months post treatment. Apart from a comparison of treatment arms, the study aimed at investigating differences between lymphoma patients and the general population and associations of HRQoL with baseline features and outcome.

## Methods

The PETAL trial (ClinicalTrials.gov no. NCT00554164) has been described previously [5, 6]. It was approved by the German Federal Institute for Drugs and Medical Devices and the ethics committees of the participating centers and performed according to the Declaration of Helsinki. All patients gave written informed consent. The first patient was included in November 2007, recruitment ended in December 2012, und the data base was locked in July 2023.

Patients aged 18–80 years with a positive baseline PET scan due to any type of aggressive non-Hodgkin lymphoma except Burkitt’s and primary central nervous system lymphoma were eligible. Total metabolic tumor volume (TMTV) was determined using the SUV_4_ method (SUV, standardized uptake value) [7, 8]. Interim PET was evaluated before treatment cycle 3 using the ΔSUV_max_ method, with a reduction ≤ 66% compared to baseline defining a positive iPET [5, 9]. Posttreatment restaging was performed 3–6 weeks after the end of therapy by computed tomography [10].

After a 3- to 7-day prephase with prednisone and vincristine, all patients were started on CHOP, combined with rituximab in patients with CD20-positive lymphoma. After two (R-)CHOP cycles, iPET-negative patients with CD20-positive lymphoma were allocated to another four cycles of R-CHOP with or without two additional doses of rituximab, while iPET-negative patients with CD20-negative lymphoma received another four cycles of CHOP. Interim PET-positive patients were randomly assigned to receive another six (R-)CHOP cycles or six blocks of an intensive methotrexate-based Burkitt’s lymphoma protocol [11]. Chemotherapy protocols and a CONSORT diagram showing recruitment and treatment allocation have been published previously [5].

HRQoL was prospectively reported by the patients using the German edition of the EORTC QLQ-C30 questionnaire, version 3.0 [12, 13]. The questionnaire was distributed by the participating centers at pretreatment staging, iPET scanning, end-of-treatment restaging and at the 3-, 6-, 9- and 12-month follow-up visits. In iPET-positive patients, an additional assessment was performed before treatment cycle 5. The EORTC QLQ-C30 is a 30-item questionnaire comprising a global quality of life scale, five functional scales (physical, role, cognitive, emotional, social) and nine symptom scales (fatigue, pain, nausea/vomiting, dyspnea, loss of appetite, insomnia, constipation, diarrhea, financial difficulties). The scales were transformed according to the scoring manual into ranges from 0 to 100 [14]. For the global quality of life and functional scales, a higher score represents a better level of function. For the symptom scales, a higher score denotes a higher level of symptoms.

The pretreatment HRQoL assessment was correlated with baseline features, treatment response and long-term outcome. Freedom from progression (FFP) was defined as the time from iPET scanning to progression; progression-free survival (PFS) was defined as the time to progression, relapse or death from any cause; and overall survival (OS) was defined as the time to death from any cause. Longitudinal HRQoL data were analyzed with regard to differences compared to baseline and differences between iPET response or treatment groups. All analyses were exploratory, applying a two-sided alpha of 0.05. Frequencies were compared using the chi^2^ test. HRQoL was compared with the reference population [15] using the one-sample t-test. For independent continuous variables, both the Mann–Whitney U/Kruskal–Wallis test and Welch’s unequal variance t-test were applied. The Mann–Whitney U/Kruskal–Wallis test was preferred for graphical display in box-plot format (box, range between the 25th and 75th percentile [interquartile range, IQR]; whiskers, data above or below the IQR within ≤ 1.5-times the extension of the IQR; circles, outliers; horizontal line, median; asterisk, mean). The Welch test was preferred for tables, because means permitted a more refined presentation of differences than medians. Paired variables were compared using the paired samples t-test. Logistic regression was used to evaluate the impact of covariates on binary variables. Time-to-event end-points were analyzed using the Kaplan–Meier estimator, the log-rank test, and, when adjusting for covariates, Cox proportional hazards regression. All authors had access to the primary clinical trial data. The analyses were carried out by UD using IBM SPSS Statistics, version 29.0, Armonk, NY, USA.

## Results

Of 862 trial participants, 558 (64.7%) completed the pretreatment HRQoL questionnaire (Figure S1). Baseline characteristics of patients with or without a pretreatment questionnaire were similar (Table S1). Binary logistic regression revealed that questionnaire completion was statistically significantly associated with male gender (odds ratio [OR] 1.412 [95% confidence interval 1.046–1.905], p = 0.024) and treatment in community hospitals (OR 3.310 [2.410–4.546], p < 0.001; as opposed to university hospitals) or private practices (OR 3.089 [1.794–5.318], p < 0.001), but not with age, International Prognostic Index (IPI) [16] or lymphoma subtype (Table S2).

### Pretreatment HRQoL in relation to an age- and gender-matched reference population

HRQoL data from randomly selected individuals from Germany were published in 2013 [15]. Compared to this population, HRQoL was inferior in trial participants, reaching statistical significance in all domains except cognitive functioning and diarrhea (Table 1). A change ≥ 10% has been shown to be relevant to the patient [17]. In female patients, a difference ≥ 10% was observed for global quality of life, role, emotional and social functioning, fatigue, dyspnea and loss of appetite. In male patients, it was observed for global quality of life, role functioning, social functioning and loss of appetite. In almost all domains, women reported worse HRQoL than men, with statistical significance for physical, role and emotional functioning, fatigue, nausea/vomiting and loss of appetite.

**Table 1:** Pretreatment quality of life in PETAL trial participants in relation to the age- and gender-matched German reference population [15]

QLQ-C30 Domain	Female				Male				Female vs male
	PETAL trial		Reference population		PETAL trial		Reference population		PETAL trial
	Median^a^	Mean^b^	Mean	p^c^	Median^a^	Mean^b^	Mean	p^c^	p^d^
Global quality of life	50.0 (33.3–66.7)	52.0 (± 25.6)	64.8	< 0.001	58.3 (33.3–75.0)	56.0 (± 24.8)	66.5	< 0.001	0.070
Functioning									
Physical	80.0 (53.3–93.3)	72.8 (± 26.2)	82.2	< 0.001	86.7 (63.3–100)	77.8 (± 24.8)	86.3	< 0.001	0.023
Role	66.7 (33.3–100)	58.1 (± 36.3)	77.1	< 0.001	66.7 (33.3–100)	65.2 (± 36.2)	80.1	< 0.001	0.026
Emotional	50.0 (33.3–75.0)	54.7 (± 26.8)	67.9	< 0.001	66.7 (41.7–83.3)	63.9 (± 26.9)	73.0	< 0.001	< 0.001
Cognitive	83.3 (66.7–100)	81.8 (± 23.4)	83.7	0.235	83.3 (66.7–100)	81.3 (± 23.1)	83.6	0.074	0.804
Social	66.7 (33.3–100)	62.6 (± 33.6)	81.0	< 0.001	66.7 (50.0–100)	65.1 (± 31.9)	82.5	< 0.001	0.385
Symptoms									
Fatigue	33.3 (22.2–66.7)	43.9 (± 30.6)	33.0	< 0.001	33.3 (11.1–55.6)	37.7 (± 29.1)	28.6	< 0.001	0.017
Nausea and vomiting	0.0 (0.0–16.7)	10.1 (± 21.6)	4.6	< 0.001	0.0 (0.0–0.0)	6.0 (± 15.9)	3.3	0.002	0.016
Pain	33.3 (0.0–66.7)	34.0 (± 34.6)	31.5	0.279	16.7 (0.0–66.7)	32.4 (± 34.6)	28.1	0.024	0.588
Dyspnea	33.3 (0.0–33.3)	30.1 (± 33.8)	18.3	< 0.001	0.0 (0.0–33.3)	24.9 (± 33.7)	16.9	< 0.001	0.077
Insomnia	33.3 (0.0–66.7)	39.8 (± 36.1)	33.6	0.011	33.3 (0.0–66.7)	37.3 (± 35.3)	25.1	< 0.001	0.415
Loss of appetite	33.3 (0.0–66.7)	31.9 (± 36.4)	8.5	< 0.001	0.0 (0.0–33.3)	21.3 (± 30.7)	7.4	< 0.001	< 0.001
Constipation	0.0 (0.0–33.3)	15.8 (± 28.8)	8.9	< 0.001	0.0 (0.0–33.3)	17.7 (± 30.2)	5.8	< 0.001	0.442
Diarrhea	0.0 (0.0–0.0)	12.1 (± 24.4)	9.3	0.087	0.0 (0.0–0.0)	10.7 (± 22.5)	9.3	0.062	0.490
Financial difficulties	0.0 (0.0–33.3)	16.7 (± 26.8)	14.0	0.143	0.0 (0.0–33.3)	19.2 (± 29.9)	13.2	< 0.001	0.300
Number of assessments	225		2,634		333		2,050		

Note that higher scores denote better performance in the global-quality-of-life and functional scales, while higher scores represent worse performance in the symptom scales

^a^ Median (interquartile range [quartile 1—quartile 3])

^b^ Mean (± standard deviation; standard deviations were not available for the reference population)

^c^ One-sample t-test (PETAL trial participants compared to reference population)

^d^ Welch test

### Association of pretreatment HRQoL with baseline features

**Fig. 1:**
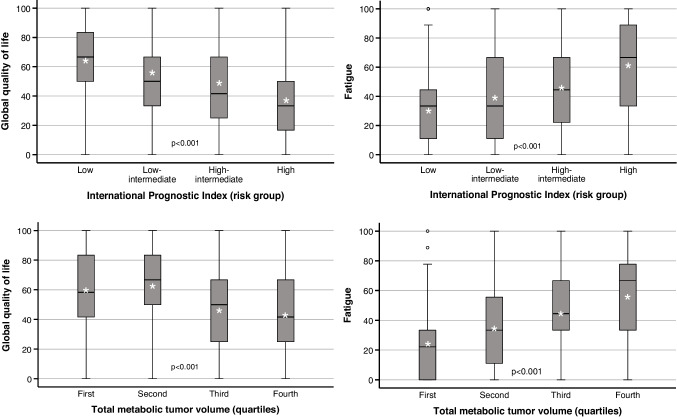
Global quality of life (left) and fatigue (right) at the pretreatment quality-of-life assessment in relation to the International Prognostic Index (top) or ascending quartiles of the total metabolic tumor volume (bottom). Cf. Table 2 for numbers of patients; p, Kruskal–Wallis test

All baseline features listed in Table S1 were significantly associated with pretreatment HRQoL (Table 2). Global quality of life, physical functioning and constipation were significantly worse in patients above age 60, while social functioning and financial difficulties were worse in younger patients. B symptoms were significantly associated with inferior performance in all domains except diarrhea and financial difficulties. With increasing risk defined by the IPI, HRQoL continuously deteriorated in all domains except diarrhea and financial difficulties. The same was observed for increasing tumor burden defined by ascending TMTV quartiles (Fig. 1).

**Table 2:** Association of pretreatment quality of life with patient baseline features

QLQ-C30 Domain			All patients		Age groups^b^			B Symptoms^b^		
			Median^a^	Mean^b^	≤ 60 years	> 60 years	p^c^	Absent	Present	p^c^
Global quality of life			50.0 (33.3–75.0)	54.4 (± 25.2)	56.8 (± 23.9)	51.6 (± 26.4)	0.015	59.5 (± 23.1)	43.3 (± 25.7)	< 0.001
Functioning										
Physical			86.7 (60.0–100)	75.8 (± 25.5)	78.1 (± 23.9)	73.2 (± 26.9)	0.025	81.1 (± 23.0)	64.3 (± 26.7)	< 0.001
Role			66.7 (33.3–100)	62.3 (± 36.3)	62.3 (± 35.9)	62.4 (± 36.9)	0.961	70.0 (± 33.2)	46.0 (± 37.3)	< 0.001
Emotional			58.3 (41.7–83.3)	60.2 (± 27.2)	58.4 (± 27.3)	62.2 (± 27.1)	0.106	63.3 (± 26.6)	53.5 (± 27.5)	< 0.001
Cognitive			83.3 (66.7–100)	81.5 (± 23.2)	83.1 (± 22.3)	79.7 (± 24.1)	0.082	84.4 (± 20.7)	74.6 (± 26.5)	< 0.001
Social			66.7 (33.3–100)	64.1 (± 32.6)	60.1 (± 32.1)	68.5 (± 32.6)	0.002	68.0 (± 30.4)	55.6 (± 35.4)	< 0.001
Symptoms										
Fatigue			33.3 (11.1–66.7)	40.2 (± 29.8)	38.8 (± 28.6)	41.8 (± 31.1)	0.238	33.2 (± 27.6)	55.1 (± 29.0)	< 0.001
Nausea and vomiting			0.0 (0.0–0.0)	7.6 (± 18.5)	7.0 (± 17.2)	8.3 (± 19.8)	0.409	4.8 (± 15.1)	13.7 (± 23.0)	< 0.001
Pain			16.7 (0.0–66.7)	33.0 (± 34.6)	33.2 (± 33.6)	32.9 (± 35.7)	0.926	29.3 (± 33.2)	41.2 (± 36.0)	< 0.001
Dyspnea			0.0 (0.0–33.3)	27.0 (± 33.8)	27.1 (± 34.1)	26.8 (± 33.6)	0.928	20.7 (± 29.9)	40.3 (± 37.8)	< 0.001
Insomnia			33.3 (0.0–66.7)	38.3 (± 35.6)	39.1 (± 35.7)	37.3 (± 35.6)	0.547	33.1 (± 33.6)	49.2 (± 37.3)	< 0.001
Loss of appetite			0.0 (0.0–50.0)	25.6 (± 33.5)	23.8 (± 31.4)	27.5 (± 35.6)	0.190	17.1 (± 28.2)	43.6 (± 36.7)	< 0.001
Constipation			0.0 (0.0–33.3)	16.9 (± 29.7)	13.9 (± 27.1)	20.3 (± 32.0)	0.012	14.2 (± 26.9)	23.0 (± 34.3)	0.003
Diarrhea			0.0 (0.0–0.0)	11.3 (± 23.2)	12.5 (± 24.2)	10.0 (± 22.1)	0.206	10.1 (± 22.4)	13.8 (± 24.8)	0.100
Financial difficulties			0.0 (0.0–33.3)	18.2 (± 28.7)	22.3 (± 31.1)	13.6 (± 25.0)	< 0.001	16.8 (± 27.9)	21.3 (± 30.2)	0.094
Number of assessments			558	558	294	264		378	179	

QLQ-C30 Domain	International Prognostic Index^b^					Total metabolic tumor volume^bd^				
	Low	Low-intermediate	High-intermediate	High	p^c^	1st quartile	2nd quartile	3rd quartile	4th quartile	p^c^
Global quality of life	63.4 (± 20.7)	54.5 (± 23.9)	47.2 (± 25.6)	36.6 (± 27.4)	< 0.001	59.9 (± 24.1)	62.3 (± 20.9)	46.5 (± 24.8)	43.3 (± 26.4)	< 0.001
Functioning										
Physical	85.2 (± 18.0)	77.0 (± 23.3)	69.5 (± 27.2)	53.7 (± 30.3)	< 0.001	86.9 (± 17.7)	81.7 (± 21.2)	70.2 (± 29.1)	60.5 (± 29.2)	< 0.001
Role	74.0 (± 29.3)	62.2 (± 35.9)	54.7 (± 37.5)	38.7 (± 40.6)	< 0.001	79.3 (± 27.3)	70.5 (± 31.7)	50.2 (± 38.2)	44.7 (± 40.2)	< 0.001
Emotional	64.5 (± 25.5)	61.1 (± 27.1)	53.1 (± 28.3)	55.3 (± 28.8)	0.002	64.4 (± 26.9)	61.8 (± 27.9)	60.3 (± 26.2)	52.4 (± 30.8)	0.037
Cognitive	85.7 (± 19.1)	83.0 (± 23.8)	76.9 (± 25.1)	72.5 (± 27.2)	< 0.001	86.6 (± 20.4)	84.0 (± 22.3)	80.0 (± 24.3)	75.1 (± 27.7)	0.009
Social	68.9 (± 28.0)	63.4 (± 32.6)	60.8 (± 36.8)	55.2 (± 37.1)	0.012	70.3 (± 29.0)	71.8 (± 29.7)	57.5 (± 33.3)	54.0 (± 37.3)	< 0.001
Symptoms										
Fatigue	30.3 (± 25.3)	39.6 (± 28.5)	47.9 (± 30.7)	60.7 (± 30.9)	< 0.001	26.2 (± 22.6)	34.9 (± 28.8)	45.9 (± 29.2)	55.8 (± 31.7)	< 0.001
Nausea and vomiting	4.3 (± 11.8)	7.2 (± 18.7)	7.9 (± 16.3)	18.2 (± 30.4)	< 0.001	2.9 (± 7.7)	5.2 (± 12.1)	12.2 (± 22.6)	15.6 (± 28.5)	< 0.001
Pain	25.8 (± 30.2)	34.0 (± 34.6)	35.0 (± 35.7)	50.4 (± 38.9)	< 0.001	19.6 (± 26.2)	29.9 (± 34.4)	43.1 (± 36.0)	42.8 (± 39.3)	< 0.001
Dyspnea	17.8 (± 28.5)	26.6 (± 31.9)	36.7 (± 37.0)	41.6 (± 39.2)	< 0.001	15.2 (± 24.9)	15.4 (± 26.1)	30.0 (± 34.5)	42.6 (± 37.4)	< 0.001
Insomnia	32.7 (± 33.4)	38.4 (± 36.4)	45.0 (± 37.1)	45.3 (± 36.2)	0.007	34.4 (± 37.0)	33.3 (± 32.6)	43.6 (± 34.8)	49.5 (± 39.2)	0.007
Loss of appetite	16.9 (± 26.8)	22.5 (± 31.4)	34.9 (± 35.7)	44.4 (± 41.5)	< 0.001	15.6 (± 25.4)	21.0 (± 30.2)	35.2 (± 36.0)	41.8 (± 39.5)	< 0.001
Constipation	10.7 (± 23.2)	16.1 (± 27.8)	22.6 (± 35.8)	29.7 (± 35.8)	< 0.001	14.1 (± 26.7)	11.9 (± 24.3)	17.2 (± 30.6)	25.5 (± 36.7)	0.025
Diarrhea	10.0 (± 22.1)	10.8 (± 22.2)	10.8 (± 21.4)	16.7 (± 29.8)	0.379	9.4 (± 20.6)	9.5 (± 20.4)	10.0 (± 22.1)	14.0 (± 26.0)	0.517
Financial difficulties	17.8 (± 27.7)	21.0 (± 30.8)	17.6 (± 29.5)	14.8 (± 26.2)	0.491	14.6 (± 25.1)	16.3 (± 29.6)	18.1 (± 27.2)	21.6 (± 32.3)	0.410
Number of assessments	230	145	108	75		92	97	89	95	

Note that higher scores denote better performance in the global-quality-of-life and functional scales, while higher scores represent worse performance in the symptom scales

^a^ Median (interquartile range [quartile 1—quartile 3])

^b^ Mean (± standard deviation)

^c^ Welch test

^d^ First quartile, 0.2–39.9 cm^3^; second quartile, 40–228.9 cm^3^; third quartile, 229–720.9 cm^3^; fourth quartile, 721–5937 cm^3^

### Association of pretreatment HRQoL with treatment response

Interim PET response was not associated with any of the HRQoL domains. End-of-treatment response was statistically significantly associated with global quality of life, physical functioning, role functioning, fatigue, dyspnea, loss of appetite and constipation (Table S3). Because statistically significant differences between complete remission, partial remission and stable disease (collectively termed controlled disease) were not observed, end-of-treatment response was dichotomized into controlled disease versus progressive disease. On binary logistic regression, the only domains retaining statistical significance when analyzed together with lymphoma subtype and IPI were physical functioning (OR per 1% increase 0.986 [0.971–1.000], p = 0.048), fatigue (OR 1.018 [1.004–1.032], p = 0.013) and loss of appetite (OR 1.015 [1.004–1.026], p = 0.007) (Table S4).

### Association of pretreatment HRQoL with long-term outcome

To assess the impact of pretreatment HRQoL on outcome, we dichotomized the EORTC QLQ C-30 scales into ‘low’ (below or equal to the median score) and ‘high’ (above the median) and investigated their association with FFP, PFS and OS (median follow-up, 10.3 years) [6].

On univariate analysis, the majority of HRQoL domains were significantly correlated with the selected end-points (Table S5). FFP was associated with global quality of life, physical, role, emotional and cognitive functioning, fatigue, dyspnea and loss of appetite. PFS was associated with global quality of life, physical, emotional and cognitive functioning, fatigue, dyspnea, insomnia and loss of appetite. OS was associated with global quality of life, physical functioning, cognitive functioning, fatigue, nausea/vomiting, dyspnea, insomnia and loss of appetite.

**Fig. 2:**
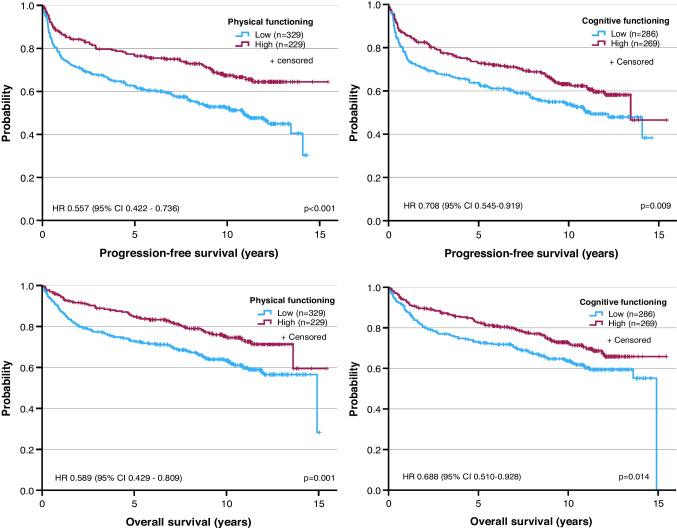
Progression-free survival (top) and overall survival (bottom) in relation to physical (left) or cognitive functioning (right) at the pretreatment quality-of-life assessment. Low, patients with scores below or equal to the median of all observed scores; high, patients with scores above the median. HR, hazard ratio; CI, confidence interval; p, log-rank test (cf. text for HR, CI and p adjusted for the covariates lymphoma subtype and International Prognostic Index)

Domains showing a significant difference between low and high scores were entered into multivariable Cox regression analyses that also included lymphoma subtype and IPI. After adjusting for these covariates, none of the scales were significantly associated with FFP. For PFS and/or OS, the only domains retaining statistical significance were physical functioning (PFS, hazard ratio [HR] 0.655 [95% confidence interval 0.486–0.883], p = 0.005; OS, HR 0.700 [0.502–0.978], p = 0.036) and cognitive functioning (PFS, HR 0.786 [0.596–1.036], p = 0.088; OS, HR 0.730 [0.534–0.997], p = 0.048) (Table S6, Fig. 2). Similar results were obtained when IPI was replaced by TMTV and age (Table S6) [7]. Treatment adherence did not differ between patients with low or high HRQoL (number of chemotherapy cycles per patient [mean ± standard deviation]: physical functioning, 6.67 ± 1.28 versus 6.75 ± 1.13, p = 0.431; cognitive functioning, 6.72 ± 1.21 versus 6.69 ± 1.23, p = 0.780).

### HRQoL during and after treatment

Of 558 patients with a pretreatment assessment, 481 completed at least one other questionnaire. There were 369 assessments at iPET scanning, 327 at the end of treatment and 265, 235, 234 and 200 at three, six, nine and 12 months of follow-up, respectively (Figure S1). Disease progression, relapse or death before the respective time-points had a statistically significant negative impact on the rate of completing the questionnaires (end of treatment: OR 0.198 [0.091–0.428], p < 0.001; 12-month follow-up, OR 0.106 [0.048–0.233], p < 0.001), while, similar to the baseline assessment, treatment in community hospitals or private practices had a positive impact (Table S2).

**Fig. 3:**
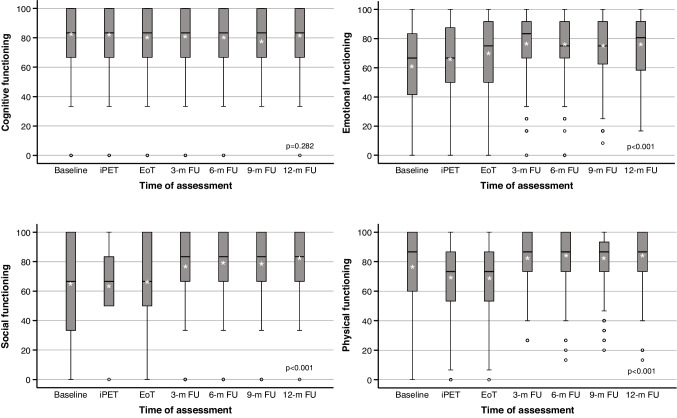
Four patterns of quality-of-life changes during treatment and follow-up. Top left, no change; top right, rapid improvement; bottom left, delayed improvement; bottom right, initial deterioration with subsequent improvement. Higher scores denote better performance. Cf. Table 3 for numbers of patients; iPET, interim positron emission tomography (before treatment cycle 3); EoT, 3–6 weeks after the end of treatment; 3-m, 6-m, 9-m and 12-m FU, 3-, 6-, 9- and 12-month follow-up; p, Kruskal–Wallis test

The longitudinal study was restricted to patients with a pretreatment questionnaire and at least one other assessment. All HRQoL domains except cognitive functioning showed statistically significant deviations from baseline (Table 3). Changes by ≥ 10% between the lowest and the highest score revealed four patterns of patient-relevant changes (Fig. 3): Domains with a stable course (< 10% change; cognitive functioning, nausea/vomiting, diarrhea, financial difficulties), domains with rapid improvement (emotional functioning, pain, insomnia, loss of appetite), domains with delayed improvement (global quality of life, social functioning) and domains with deterioration followed by improvement (physical functioning, role functioning, fatigue, dyspnea, constipation). Three months after the end of treatment, almost all HRQoL domains had returned to levels similar to the reference population (Table S7). The only domain to differ by > 10% was pain (lower in PETAL trial participants).

**Table 3:** Quality-of-life changes during treatment and follow-up

QLQ-C30 Domain	Baseline		Interim PET		End of treatment		3-month follow-up		6-month follow-up		9-month follow-up		12-month follow-up	
	Median^a^	Mean^b^	Mean^b^	p^c^	Mean^b^	p^c^	Mean^b^	p^c^	Mean^b^	p^c^	Mean^b^	p^c^	Mean^b^	p^c^
Global quality of life	58.3 (33.3–75.0)	55.6 (± 24.6)	57.9 (± 21.5)	0.142	60.7 (± 21.1)	0.002	70.5 (± 19.2)	< 0.001	71.8 (± 20.6)	< 0.001	70.7 (± 20.5)	< 0.001	72.9 (± 18.5)	< 0.001
Functioning														
Physical	86.7 (60.0–100)	77.4 (± 25.0)	69.3 (± 23.2)	< 0.001	69.6 (± 23.2)	< 0.001	82.0 (± 18.0)	0.003	83.2 (± 17.3)	< 0.001	81.7 (± 17.8)	0.008	83.6 (± 17.7)	< 0.001
Role	66.7 (33.3–100)	63.8 (± 35.7)	54.8 (± 31.6)	< 0.001	55.8 (± 30.0)	< 0.001	72.5 (± 25.8)	< 0.001	75.1 (± 25.9)	< 0.001	74.5 (± 26.6)	< 0.001	76.0 (± 25.3)	< 0.001
Emotional	66.7 (41.7–83.3)	60.8 (± 27.2)	67.4 (± 24.4)	< 0.001	68.9 (± 23.5)	< 0.001	76.6 (± 21.1)	< 0.001	75.0 (± 22.2)	< 0.001	74.5 (± 22.4)	< 0.001	75.5 (± 21.9)	< 0.001
Cognitive	83.3 (66.7–100)	82.4 (± 22.8)	82.0 (± 22.5)	0.789	80.3 (± 23.9)	0.209	80.9 (± 22.0)	0.374	80.1 (± 23.7)	0.222	78.2 (± 23.7)	0.027	81.1 (± 22.1)	0.509
Social	66.7 (33.3–100)	64.8 (± 32.2)	63.9 (± 28.6)	0.650	65.4 (± 30.1)	0.806	77.5 (± 24.8)	< 0.001	79.8 (± 24.4)	< 0.001	79.2 (± 24.5)	< 0.001	82.2 (± 21.5)	< 0.001
Symptoms														
Fatigue	33.3 (11.1–66.7)	38.9 (± 29.4)	47.7 (± 26.5)	< 0.001	45.1 (± 27.2)	0.002	29.4 (± 23.1)	< 0.001	29.1 (± 23.0)	< 0.001	31.3 (± 24.0)	< 0.001	29.1 (± 23.4)	< 0.001
Nausea and vomiting	0.0 (0.0–0.0)	7.4 (± 18.1)	10.1 (± 17.7)	0.026	7.2 (± 17.3)	0.914	2.9 (± 8.8)	< 0.001	3.0 (± 10.0)	< 0.001	3.9 (± 11.1)	0.002	3.5 (± 11.3)	< 0.001
Pain	16.7 (0.0–66.7)	31.6 (± 34.1)	23.8 (± 28.4)	< 0.001	19.3 (± 26.6)	< 0.001	16.4 (± 24.0)	< 0.001	16.7 (± 25.5)	< 0.001	17.2 (± 24.4)	< 0.001	15.3 (± 23.1)	< 0.001
Dyspnea	0.0 (0.0–33.3)	24.8 (± 32.4)	26.0 (± 31.1)	0.563	29.0 (± 30.5)	0.058	17.2 (± 24.0)	< 0.001	16.9 (± 25.0)	< 0.001	19.0 (± 24.6)	0.008	20.1 (± 24.8)	0.043
Insomnia	33.3 (0.0–66.7)	36.6 (± 34.9)	35.3 (± 32.3)	0.578	33.8 (± 34.7)	0.267	25.6 (± 29.9)	< 0.001	27.4 (± 30.3)	< 0.001	27.8 (± 31.4)	< 0.001	28.2 (± 29.7)	0.001
Loss of appetite	0.0 (0.0–33.3)	23.4 (± 32.2)	24.3 (± 32.0)	0.710	20.3 (± 30.8)	0.166	8.1 (± 18.9)	< 0.001	6.9 (± 18.3)	< 0.001	8.2 (± 20.4)	< 0.001	8.1 (± 18.8)	< 0.001
Constipation	0.0 (0.0–33.3)	16.8 (± 29.7)	20.7 (± 30.8)	0.069	13.5 (± 25.7)	0.094	8.6 (± 20.4)	< 0.001	8.5 (± 20.1)	< 0.001	10.3 (± 21.9)	0.001	9.4 (± 21.0)	< 0.001
Diarrhea	0.0 (0.0–0.0)	11.3 (± 23.6)	11.4 (± 22.7)	0.934	12.3 (± 24.2)	0.595	7.7 (± 20.0)	0.031	7.3 (± 17.7)	0.011	7.7 (± 19.0)	0.035	4.9 (± 14.4)	< 0.001
Financial difficulties	0.0 (0.0–33.3)	18.2 (± 28.8)	24.2 (± 31.4)	0.005	26.4 (± 32.3)	< 0.001	19.0 (± 29.9)	0.707	18.1 (± 28.9)	0.914	19.2 (± 29.1)	0.676	15.6 (± 24.4)	0.237
Number of assessments	481		369		327		265		235		234		200	

Note that higher scores denote better performance in the global-quality-of-life and functional scales, while higher scores represent worse performance in the symptom scales

^a^ Median (interquartile range [quartile 1—quartile 3])

^b^ Mean (± standard deviation)

^c^ Compared to baseline assessment (Welch test)

Because many patients missed one or several assessments, different points in time were represented by different patient groups. To counteract a selection bias, we performed an additional analysis in 82 patients with complete longitudinal HRQoL data. The above results were confirmed and the same four patterns of changes emerged (Table S8).

### Impact of type of chemotherapy, iPET response and end-of-treatment response on HRQoL

Twenty-seven iPET-negative patients with CD20-negative lymphoma received six cycles of CHOP, 224 and 174 iPET-negative patients with CD20-positive lymphoma received six cycles of R-CHOP or six cycles of R-CHOP followed by two doses of rituximab, and 30 and 26 iPET-positive patients received eight cycles of (R-)CHOP or two cycles of (R-)CHOP followed by six cycles of the Burkitt protocol (rituximab restricted to CD20-positive lymphoma). In the iPET-negative group, no consistent HRQoL differences were observed between six cycles of R-CHOP with or without two extra doses of rituximab (Table S9). Likewise, in the iPET-positive group, there were no consistent differences between eight cycles of (R-)CHOP and two cycles of (R-)CHOP followed by the Burkitt protocol (Table S10). Because of small numbers, these analyses could not be repeated in patients with complete longitudinal data.

**Fig. 4:**
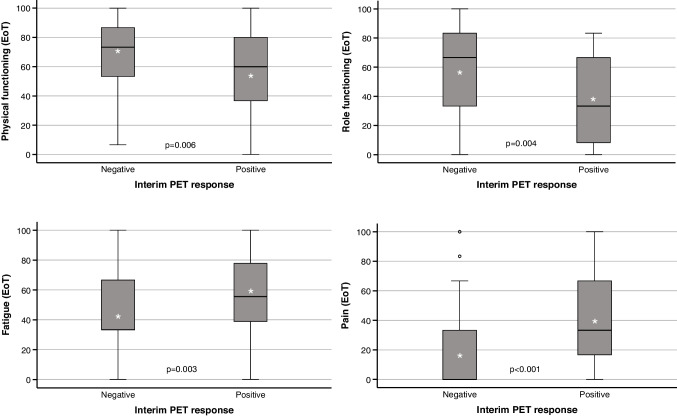
Functioning (top) and symptoms (bottom) at the end-of-treatment (EoT) quality-of-life assessment in relation to the interim PET response. Negative interim PET response, 299 patients; positive interim PET response, 28 patients. Note that higher scores denote better performance in the functional scales, while higher scores represent worse performance in the symptom scales. p, Kruskal–Wallis test

The only point in time to show statistically significant HRQoL differences between iPET-negative and iPET-positive patients was the end-of-treatment assessment where the domains physical, role, emotional and social functioning, fatigue, pain and loss of appetite showed significantly inferior results in iPET-positive patients (Fig. 4, Table S11). This was associated with a significantly higher proportion of iPET-positive patients experiencing disease progression despite therapy (5/27 versus 2/298; p < 0.001). Patients with progressive disease reported significantly worse global quality of life, physical functioning, social functioning, fatigue, pain and dyspnea (Table 4). Even within the same end-of-treatment response group, iPET-positive patients tended to report lower HRQoL than iPET-negative patients, but most of the differences failed to reach statistical significance (Table S12).

**Table 4:** Quality of life at the end of treatment in relation to the remission status

QLQ-C30 domain	End-of-treatment assessment^a^				
	Complete remission	Partial remission	Stable disease	Progressive disease	p^b^
Global quality of life	60.5 (± 20.6)	64.1 (± 19.8)	68.3 (± 22.4)	31.0 (± 22.4)	0.024
Functioning					
Physical	70.6 (± 21.9)	70.1 (± 24.0)	70.7 (± 19.2)	33.6 (± 28.0)	0.045
Role	56.2 (± 29.6)	56.7 (± 30.5)	66.7 (± 26.4)	33.3 (± 34.7)	0.365
Emotional	67.5 (± 24.5)	74.1 (± 19.6)	65.0 (± 16.0)	56.0 (± 27.1)	0.113
Cognitive	79.3 (± 23.8)	83.5 (± 22.7)	73.3 (± 34.6)	78.6 (± 32.9)	0.586
Social	65.7 (± 30.1)	67.9 (± 28.5)	56.7 (± 32.5)	25.0 (± 27.4)	0.032
Symptoms					
Fatigue	44.3 (± 26.9)	44.0 (± 26.2)	42.2 (± 27.7)	82.5 (± 27.1)	0.031
Nausea and vomiting	5.6 (± 14.1)	8.8 (± 19.1)	10.0 (± 9.1)	42.9 (± 46.0)	0.149
Pain	20.2 (± 26.0)	14.1 (± 23.9)	3.3 (± 7.5)	61.9 (± 43.8)	0.002
Dyspnea	31.2 (± 30.4)	21.8 (± 28.8)	13.3 (± 18.3)	52.4 (± 42.4)	0.047
Insomnia	34.9 (± 34.6)	28.6 (± 33.0)	20.0 (± 18.3)	57.1 (± 53.5)	0.196
Loss of appetite	18.7 (± 29.1)	22.2 (± 34.7)	26.7 (± 27.9)	42.9 (± 37.1)	0.377
Constipation	14.0 (± 25.6)	11.3 (± 25.1)	6.7 (± 14.9)	33.3 (± 38.5)	0.394
Diarrhea	12.5 (± 24.6)	10.8 (± 20.4)	0.0 (± 0.0)	33.3 (± 47.1)	0.342
Financial difficulties	24.4 (± 30.8)	30.8 (± 34.3)	26.7 (± 27.9)	42.9 (± 53.5)	0.495
Number of assessments	235	78	5	7	

Note that higher scores denote better performance in the global-quality-of-life and functional scales, while higher scores represent worse performance in the symptom scales. The analysis was restricted to 325 (instead of 327) patients because the remission status of two patients was not reported

^a^ Mean (± standard deviation)

^b^ Welch test (substituted by Mann–Whitney U test in case of 0 variance)

### Impact of observation time on patient selection

**Fig. 5:**
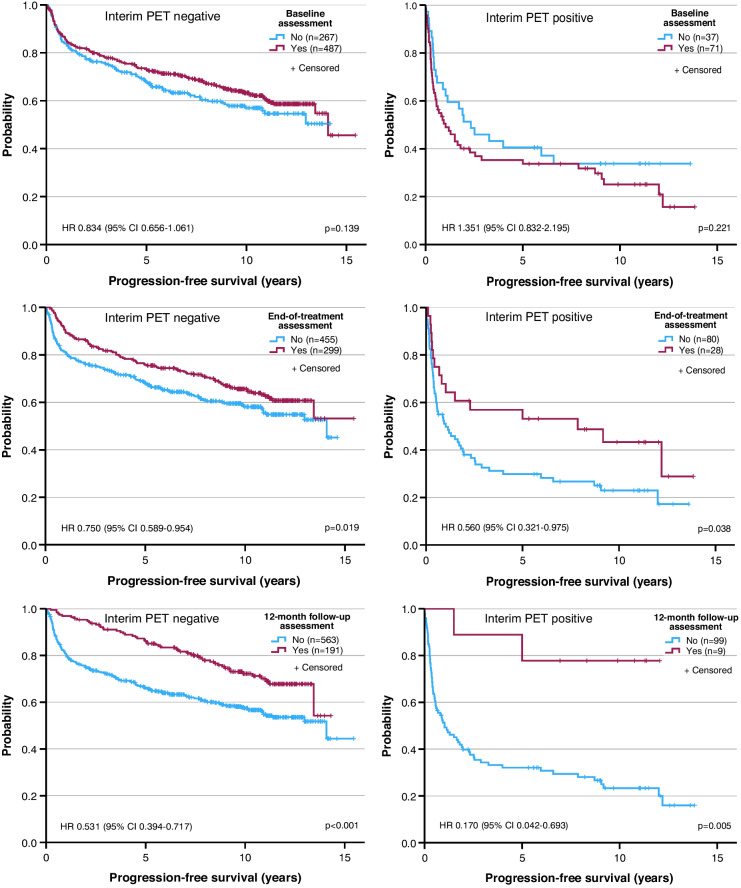
Progression-free survival in relation to completion versus non-completion of the quality-of-life questionnaire before treatment (top), at end of treatment (middle) and after 12 months of follow-up (bottom). Left, interim PET negative patients (good prognosis); right, interim PET positive patients (poor prognosis). HR, hazard ratio; CI, confidence interval; p, log-rank test

The detrimental effect of iPET positivity on HRQoL was only observed at the end of treatment, not during follow-up. This raised the question of whether the patient groups completing the questionnaires at different times were comparable. To investigate patient selection, the population was split at baseline, end of treatment and 12-months of follow-up with regard to completion versus non-completion of the questionnaire, and the PFS of the two groups was compared at each time. With increasing time from diagnosis, the outcome of the population that completed the questionnaires continuously improved (Fig. 5). This was more pronounced among iPET-positive patients who had a high risk of terminating the study early due to progression, relapse or death, than among iPET-negative patients where adverse outcomes were rare.

### HRQoL in diffuse large B-cell lymphoma

Because of low patient numbers in less common lymphoma subtypes (Table S1), the subgroup analysis was restricted to diffuse large B-cell lymphoma (400 pretreatment questionnaires). Although some comparisons failed to reach statistical significance, the results described for the entire population were reproduced in the subgroup (Tables S13-S18, Figures S2 and S3).

## Discussion

The most important results of our HRQoL study can be summarized as follows: First, pretreatment HRQoL was associated with baseline patient features, end-of-treatment response and long-term outcome. Second, individual HRQoL domains differed in their response to lymphoma treatment. Third, comparisons of randomized treatment arms were hampered by small patient numbers and decreasing participation with increasing time from diagnosis. And fourth, end-of-treatment HRQoL was better in iPET-negative patients than in iPET-positive patients.

In the German reference population, women reported a worse HRQoL than men, and HRQoL deteriorated with increasing age [15]. These findings were reproduced among PETAL trial participants, although pretreatment HRQoL was significantly worse when compared to the reference population. In contrast to the reference population, older patients reported better social functioning and less financial difficulties than young patients. This is in line with a recent report on German blood cancer survivors where financial problems were significantly more frequent among young patients [18].

We confirmed findings from smaller studies indicating that pretreatment HRQoL is associated with B symptoms and IPI [3, 4, 19]. We have previously shown that all IPI factors except age are surrogates for tumor burden [7]. In the present study, TMTV, a direct measure of tumor burden, was tightly associated with almost all HRQoL domains.

In line with previous reports [2, 19, 20], some pretreatment HRQoL domains predicted PFS and OS independent of the IPI. Importantly, none of the scales predicted FFP. In contrast to PFS and OS, FFP does not include death from any cause. It best reflects natural disease history and treatment efficacy [7]. Failure to predict FFP implies that prediction of PFS and OS by physical and cognitive functioning was predominantly driven by death from any cause. The life expectancy of individuals with self-reported disability is reduced, in particular in the presence of motor impairment, physical illness or mental health problems including mild cognitive impairment [21, 22]. Thus, in addition to impairment due to lymphoma, shorter survival of patients with low physical or cognitive functioning may have been related to pre-existing comorbidities.

Our longitudinal study revealed characteristic HRQoL changes during treatment and follow-up. Some of these have also been observed in smaller studies [3, 4, 20, 23]. Rapid improvements soon after the start of chemotherapy were seen for emotional functioning, pain, insomnia and loss of appetite. This mirrors the clinical experience that patients feel better, sleep better and eat better as soon as the uncertainty of how to deal with the disease is overcome. The administration of prednisone during prephase and R-CHOP therapy may also have had a positive effect on the patients’ well-being [24]. Less pain may be attributed to therapy-induced tumor shrinkage. Global quality of life and social functioning remained stable during chemotherapy and improved after its completion. Physical and role functioning, fatigue, dyspnea and constipation deteriorated during treatment. Chemotherapy-induced anemia can worsen physical performance, fatigue and dyspnea [3]. Constipation is a frequent side effect of vinca alkaloids, analgesics and antiemetics [25]. Treatment-related loss of available time may impede role functioning. Cognitive functioning, nausea/vomiting, diarrhea and financial difficulties remained constant throughout the study period. At no point in time was cognitive functioning impaired by disease or treatment, and antiemetics efficiently prevented chemotherapy-induced nausea and vomiting. During follow-up, HRQoL did not substantially differ from the reference population.

One of the reasons to perform the present study was the expectation that a switch from R-CHOP to the Burkitt protocol would result in worse HRQoL. We previously reported that adverse events, in particular infections and mucositis, were significantly more frequent among patients on the Burkitt protocol than among patients on R-CHOP [5]. Although the patients’ self-reported HRQoL appeared similar in the two treatment arms, small patient numbers precluded a reliable comparison. No HRQoL differences between randomized treatments were seen in elderly lymphoma patients receiving R-CHOP with or without granulocyte colony-stimulating factor [4] or modified CHOP versus an elderly-specific regimen [3]. By contrast, in a randomized comparison of CHOP and MACOP-B, global quality of life, physical functioning and fatigue were significantly worse in patients on MACOP-B [1]. MACOP-B and the Burkitt protocol used in our study have a similar composition, both including high-dose methotrexate as a major constituent [11]. The selection of patients, however, differed in the two studies. In the MACOP-B trial, all patients underwent randomization, whereas, in our trial, randomization was restricted to patients failing to respond to the initial cycles of chemotherapy. Thus, in addition to potential improvements in supportive care and with the caveat of small patient numbers, knowledge of impending treatment failure may have had a stronger effect on HRQoL than treatment with a more intensive regimen.

Patients with progressive disease reported worse HRQoL at end of treatment than patients responding to therapy. Since progressive disease was more frequent among iPET-positive patients than among iPET-negative patients, it was not surprising that the former reported worse HRQoL than the latter. However, even within the same end-of-treatment response group, HRQoL appeared worse in iPET-positive patients. This may have been related to longer chemotherapy duration (eight versus six cycles) and fear of treatment failure.

Patients from community hospitals and private practices completed the HRQoL questionnaires significantly more often than patients from university hospitals. German university hospitals are characterized by a high proportion of physicians in training [26] who are less experienced not only in patient care, but also in the conduct of clinical trials. Their curriculum involves frequent rotations that interrupt the continuity of patient care. In line with this, hematological patients from university hospitals complained more often about frequently changing physicians than patients from other institutions [27]. A high proportion of inexperienced physicians and frequently changing treating physicians may explain the limited contribution of university hospitals to the HRQoL part of the PETAL trial.

Strengths of our study include high patient numbers and standardized diagnostic and therapeutic procedures. Its major weakness is attrition of participation during treatment and follow-up. At baseline, the HRQoL participation rate in the PETAL trial (64.7%) was similar to other randomized non-Hodgkin lymphoma trials (64.2–77.6%) [1–4]. At the end of treatment and follow-up, however, participation was considerably lower (37.9% versus 45.3–66.0%; 23.2% versus 38.3–54.7%). Differences in trial design and logistics may explain these findings. In the PETAL trial, all patients were eligible for the HRQoL assessment, while, in the other studies, the assessment was restricted to a limited number of selected sites [1–4]. In addition, in the PETAL trial, the participating institutions were solely responsible for handling the questionnaires. In the other trials, the questionnaires were completed with the help of a study nurse [3] or mailed at the respective times by the central trial office to the patients’ personal address [1, 2, 4]. This may have helped maintain a high participation rate.

All in all, less than 10% of patients submitted a complete set of HRQoL questionnaires. Thus, in most analyses, different time-points were represented by different patients. Because progression, relapse or death led to premature discontinuation of the HRQoL study, the remaining patients’ outcome improved with increasing study duration. A similar positive selection has been observed in other longitudinal cancer studies. Apart from adverse health outcomes, these studies identified low socioeconomic status, unhealthy life-style, comorbidity, female gender and age below 60 or above 70 as risk factors for non-response to questionnaires [28, 29]. Our study demonstrates that the extent of positive selection depends on the prognosis: The worse the overall prognosis, the more participants drop out of the study prematurely and the better the prognosis of the remaining participants.

In conclusion, self-reported HRQoL was correlated with tumor burden and survival. The response to therapy differed among HRQoL domains, but all domains returned to normal during follow-up. Although no differences were observed between individual treatment protocols, the longitudinal data must be interpreted with caution because of small numbers and a selection bias related to progression, relapse, death and other factors.

## Supplementary Information

Below is the link to the electronic supplementary material.

Supplementary Material 1 (PDF 0.99 MB)

## Data Availability

The datasets generated and analyzed during this study are available from the corresponding author on reasonable request.
