# Supplementary material for: Health-related quality of life in patients with aggressive non-Hodgkin lymphoma: results from the PETAL trial
Source: Ann Hematol. 2025 May 21;104(5):2831–45. doi: 10.1007/s00277-025-06402-1 (PMC12141138; doi:10.1007/s00277-025-06402-1)
Supplement: Supplementary file 1 — Supplementary Material 1 (PDF 0.99 MB) [file 277_2025_6402_MOESM1_ESM.pdf]

Supplementary Information (Annals of Hematology)

Health-related quality of life in patients with aggressive non-Hodgkin lymphoma: Results from the PETAL trial

Ulrich Dührsen, Gabriele Prange-Krex, Regina Moeller, Harald Held, Gerhard Heil, Andreas Schwarzer, Stefan Mahlmann, Ariane Dienst, Matthias Sandmann, Georg Maschmeyer, Jochen Schütte, Dennis Hahn, Michael Heike, Michael Nonnemacher, Christine Hanoun, Andreas Hüttmann, on behalf of the PETAL trial investigators

Corresponding author:
Prof. Dr. Ulrich Dührsen, MD
University Hospital of Essen, Germany
Email: ulrich.duehrsen@uk-essen.de

1. Supplemental Tables Page
Supplemental Table S1 2
Supplemental Table S2 4
Supplemental Table S3 6
Supplemental Table S4 7
Supplemental Table S5 9
Supplemental Table S6 10
Supplemental Table S7 14
Supplemental Table S8 16
Supplemental Table S9 17
Supplemental Table S10 19
Supplemental Table S11 21
Supplemental Table S12 22
Supplemental Table S13 23
Supplemental Table S14 25
Supplemental Table S15 26
Supplemental Table S16 29
Supplemental Table S17 30
Supplemental Table S18 31

2. Supplemental Figures
Supplemental Figure S1 32
Supplemental Figure S2 33
Supplemental Figure S3 34

3. Full list of sites and investigators 35

1. Supplemental Tables

Table S1. Features of patients with or without a pretreatment quality-of-life assessment

**Parameter**	**Pretreatment assessment**	**No pretreatment assessment**	**p[a]**
Number of patients	558	304	n.a.
Type of lymphoma			
Diffuse large B-cell lymphoma	400 (71.7)	223 (73.4%)	0.978
Primary mediastinal B-cell lymphoma	30 (5.4)	12 (3.9)	
Follicular lymphoma grade 3a or 3b	36 (6.5)	18 (5.9)	
Other aggressive B-cell lymphoma[b]	27 (4.8)	16 (5.3)	
ALK+ anaplastic large cell lymphoma	13 (2.3)	8 (2.6)	
ALK- peripheral T-cell lymphoma[c]	36 (6.5)	19 (6.3)	
Other diagnosis[d]	16 (2.8)	8 (2.6)	
Age (years)			
Median (range)	59 (18-80)	61 (18-80)	0.098[e]
Age group			
≤ 60 years	294 (52.7)	149 (49.0)	0.302
> 60 years	264 (47.3)	155 (51.0)	
Gender			
Female	225 (40.3)	150 (49.3)	0.011
Male	333 (59.7)	154 (50.7)	
International Prognostic Index (risk group)[f]			
Low	230 (41.2)	99 (33.0)	0.077
Low-intermediate	145 (26.0)	79 (26.3)	
High-intermediate	108 (19.4)	72 (24.0)	
High	75 (13.4)	50 (16.7)	
B symptoms[g]			
No	378 (67.9)	214 (71.6)	0.263
Yes	179 (32.1)	85 (28.4)	
Total metabolic tumor volume (cm^3)[h]			
Median (range)	225 (0-5936)	244 (0-5111)	0.643
Interim PET response			
Negative (>66% SUV_{max} reduction)	487	267	0.815
Positive (≤66% SUV_{max} reduction)	71	37	

End-of-treatment response[i]			
Complete remission	359	188	0.415
Partial remission	128	69	
Stable disease	18	16	
Progressive disease	31	21	
Outcome[j]			
Freedom from progression at 5 years	75.1 (71.4-78.8)	73.3 (68.2-78.4)	0.914
Progression-free survival at 5 years	68.5 (64.6-72.4)	64.3 (58.8-69.8)	0.367
Overall survival at 5 years	77.7 (74.2-81.2)	71.7 (66.6-76.8)	0.086
Treatment institution			
University hospital (n=15)	193	187	<0.001
Community hospital (n=28)	298	95	
Private practice (n=12)	67	22	

Numbers are given as n (%) unless otherwise noted. ALK+, anaplastic lymphoma kinase-positive; ALK-, ALK-negative; SUV_{max}, maximum standardized uptake value

[a] chi^2 test, unless otherwise noted

[b] Intermediate between diffuse large B-cell lymphoma and Burkitt lymphoma, 3: intermediate between diffuse large B-cell lymphoma and Hodgkin lymphoma, 3; unclassified aggressive B-cell lymphoma, 18

[c] Peripheral T-cell lymphoma, not otherwise specified, 20; angioimmunoblastic T-cell lymphoma, 18; ALK-negative anaplastic large cell lymphoma, 13; unclassified T-cell lymphoma, 4

[d] Hodgkin lymphoma, 1; Burkitt lymphoma, 2; mantle cell lymphoma, 2; indolent B-cell lymphoma, 18; breast cancer, 1

[e] Mann-Whitney U test

[f] Data from only 858 patients available

[g] Data from only 856 patients available

[h] Data from only 567 patients available

[i] Data from only 830 patients available

[j] Kaplan-Meier estimate of the percentage of patients without an event after 5 years (95% confidence interval)

Table S2. Binary logistic regression analysis of factors with potential impact on completing the health-related quality of life questionnaire

Variable level	Odds ratio[a]	p[b]
**Baseline questionnaire**		
**Gender**[c]		
Male	1.412 (1.046-1.905)	0.024
**Age** (continuous variable)		
Per year	0.991 (0.979-1.002)	0.112
**International Prognostic Index**[d]		
Low intermediate risk	0.774 (0.528-1.136)	0.190
High-intermediate risk	0.704 (0.466-1.065)	0.097
High risk	0.741 (0.458-1.199)	0.222
**Lymphoma subtype**[e]		
Primary mediastinal B-cell lymphoma	1.434 (0.672-3.060)	0.351
Follicular lymphoma grade 3	1.014 (0.545-1.888)	0.965
Other aggressive B-cell lymphoma	1.068 (0.541-2.109)	0.850
ALK+ anaplastic large cell lymphoma	0.685 (0.259-1.810)	0.446
ALK- peripheral T-cell lymphoma	1.025 (0.557-1.885)	0.938
Other diagnosis	1.950 (0.715-5.320)	0.192
**Treatment institution**[f]		
Community hospital	3.310 (2.410-4.546)	<0.001
Private practice	3.089 (1.794-5.318)	<0.001
**End-of-treatment questionnaire**		
**Gender**[c]		
Male	1.294 (0.968-1.730)	0.082
**Interim PET response**[g]		
Positive (unfavorable)	0.691 (0.424-1.125)	0.137
**Progression or death before the end of treatment**[h]		
Yes	0.198 (0.091-0.428)	<0.001
**Treatment institution**[f]		
Community hospital	1.896 (1.397-2.574)	<0.001
Private practice	3.583 (2.206-5.820)	<0.001

Questionnaire at 12 months of follow-up		
**Gender**[c]		
Male	1.115 (0.793-1.567)	0.531
**Interim PET response**[g]		
Positive (unfavorable)	0.425 (0.203-0.886)	0.022
**Progression or death before 12 months of follow-up**[h]		
Yes	0.106 (0.048-0.233)	<0.001
**Treatment institution**[f]		
Community hospital	1.543 (1.070-2.227)	0.020
Private practice	4.602 (2.732-7.753)	<0.001

Similar results were obtained when age was treated as a dichotomous variable (≤ 60 years versus > 60 years).
ALK+, anaplastic lymphoma kinase-positive; ALK-, ALK-negative
Nagelkerke's R^2 (top to bottom), 0.119 – 0.105 – 0.182
[a] Odds ratio (95% confidence interval)
[b] Wald test
[c] Reference, female
[d] Reference, low risk
[e] Reference, diffuse large B-cell lymphoma
[f] Reference, university hospital
[g] Reference, negative (favorable)
[h] Reference, no progression or death (end of treatment: progression without death 52 patients, progression with lymphoma-related death 2 patients, death unrelated to lymphoma progression 20 patients; 12 months of follow-up: progression without death 74 patients, progression with lymphoma-related death 80 patients, death unrelated to lymphoma progression 31 patients)

Table S3. Association of pretreatment quality of life with interim and end-of-treatment response

QLQ-C30 Domain	Interim PET response[a]			End-of treatment response[a]				
	Favorable	Unfavorable	p[b]	Complete remission	Partial remission	Stable disease	Progressive disease	p[b]
**Global quality of life**	54.5 (±25.0)	53.4 (±26.5)	0.746	56.2 (±24.1)	53.1 (±25.4)	58.8 (±27.4)	39.2 (± 27.2)	0.012
**Functioning**								
Physical	76.2 (±24.8)	72.8 (±29.9)	0.361	79.4 (±22.5)	71.8 (±27.8)	74.4 (±29.4)	58.1 (± 31.7)	< 0.001
Role	62.5 (±36.1)	61.0 (±38.0)	0.742	66.7 (±34.5)	56.0 (±38.4)	58.3 (±35.8)	43.0 (±40.3)	0.003
Emotional	60.3 (±27.2)	59.3 (±28.0)	0.767	60.7 (±27.1)	60.0 (±26.9)	63.7 (±27.6)	53.2 (±28.3)	0.528
Cognitive	81.6 (±23.1)	80.8 (±23.8)	0.768	82.7 (±21.7)	82.2 (±24.4)	80.4 (±27.8)	72.6 (±27.4)	0.278
Social	65.1 (±32.2)	57.4 (±34.6)	0.083	65.4 (±31.9)	64.3 (±32.0)	59.8 (±36.8)	51.1 (±37.5)	0.233
**Symptoms**								
Fatigue	40.2 (±29.3)	40.4 (±33.6)	0.965	37.4 (±28.5)	42.1 (±30.3)	42.0 (±32.7)	60.2 (± 32.0)	0.003
Nausea and vomiting	7.3 (±18.2)	9.9 (±20.0)	0.317	6.6 (±16.0)	7.9 (±20.4)	9.3 (±24.4)	15.6 (±24.3)	0.235
Pain	32.9 (±34.4)	34.3 (±35.6)	0.754	32.2 (±33.9)	34.9 (±34.6)	22.2 (±29.1)	39.8 (±37.9)	0.283
Dyspnea	27.2 (±33.7)	25.8 (±34.8)	0.763	23.7 (±31.9)	31.0 (±35.3)	33.3 (±39.1)	43.0 (±39.6)	0.023
Insomnia	38.8 (±35.4)	34.7 (±37.1)	0.390	36.3 (±34.9)	40.2 (±37.9)	40.7 (±31.4)	44.1 (±35.9)	0.542
Loss of appetite	25.2 (±33.4)	28.2 (±34.6)	0.495	23.2 (±32 6)	24.2 (±32.1)	22.2 (±30.2)	50.5 (±38.4)	0.004
Constipation	16.7 (±29.2)	18.8 (±32.7)	0.608	17.5 (±30.3)	13.0 (±26.3)	6.3 (±18.1)	27.1 (±34.3)	0.024
Diarrhea	11.0 (±23.2)	13.1 (±23.6)	0.473	11.2 (±23.6)	10.7 (±22.5)	10.4 (±20.1)	17.2 (±28.4)	0.702
Financial difficulties	17.7 (±28.6)	21.4 (±29.5)	0.328	18.1 (±29.0)	21.0 (±29.6)	14.6 (±29.7)	11.1 (±25.3)	0.314
**Number of assessments**	487	71		359	128	18	32	

Note that higher scores denote better performance in the global-quality-of-life and functional scales, while higher scores represent worse performance in the symptom scales.
[a] Mean (± standard deviation)
[b] Welch test

Table S4. Binary logistic regression analysis of baseline factors with potential impact on disease progression at end of treatment

**Variables**<br>**Lymphoma subtype, International Prognostic Index, quality of life**			**Variables**<br>**Age group, lymphoma subtype, total metabolic tumor volume, quality of life**		
**Variable level**	**Odds ratio[a]**	**p[b]**	**Variable level**	**Odds ratio[a]**	**p[b]**
**Lymphoma subtype[c]**			**Age group[d]**		
Primary mediastinal B-cell lymphoma	0.000[e]	0.998	> 60 years	0.832 (0.319-2.171)	0.707
Follicular lymphoma grade 3	0.000[e]	0.998	**Lymphoma subtype[c]**		
ALK+ anaplastic large cell lymphoma	0.000[e]	0.999	ALK+ anaplastic large cell lymphoma	0.000[e]	0.999
ALK- peripheral T-cell lymphoma	5.173 (1.937-13.810)	0.001	ALK- peripheral T-cell lymphoma	9.842 (3.182-30.445)	< 0.001
**International Prognostic Index[f]**			**Total metabolic tumor volume[g]**		
Low-intermediate risk	5.617 (1.115-28.302)	0.036	Second quartile	0.528 (0.045-6.216)	0.611
High-intermediate risk	9.984 (2.095-47.581)	0.004	Third quartile	4.575 (0.807-25.947)	0.086
High risk	7.984 (1.474-43.250)	0.016	Fourth quartile	9.084 (1.782-46.311)	0.008
**Physical functioning**			**Physical functioning**		
Continuous variable (per 1% increase)	0.986 (0.971-1.000)	0.048	Continuous variable (per 1% increase)	0.993 (0.978-1.009)	0.414
**Lymphoma subtype[c]**			**Age group[d]**		
Primary mediastinal B-cell lymphoma	0.000[e]	0.998	> 60 years	0.843 (0.321-2.219)	0.730
Follicular lymphoma grade 3	0.000[e]	0.998	**Lymphoma subtype[c]**		
ALK+ anaplastic large cell lymphoma	0.000[e]	0.999	ALK+ anaplastic large cell lymphoma	0.000[e]	0.999
ALK- peripheral T-cell lymphoma	5.824 (2.153-15.755)	< 0.001	ALK- peripheral T-cell lymphoma	9.977 (3.205-31.054)	< 0.001
**International Prognostic Index[f]**			**Total metabolic tumor volume[g]**		
Low-intermediate risk	5.898 (1.156-30.083)	0.033	Second quartile	0.508 (0.043-6.031)	0.592
High-intermediate risk	9.966 (2.088-47.580)	0.004	Third quartile	4.117 (0.716-23.683)	0.113
High risk	8.414 (1.580-44.802)	0.013	Fourth quartile	8.084 (1.565-41.761)	0.013

**Fatigue**			**Fatigue**		
Continuous variable (per 1% increase)	1.018 (1.004-1.032)	0.013	Continuous variable (per 1% increase)	1.011 (0.996-1.026)	0.166
**Lymphoma subtype**[c]			**Age group**[d]		
Primary mediastinal B-cell lymphoma	0.000[le]	0.998	> 60 years	0.806 (0.307-2.118)	0.662
Follicular lymphoma grade 3	0.000[e]	0.998	**Lymphoma subtype**[c]		
ALK+ anaplastic large cell lymphoma	0.000[e]	0.999	ALK+ anaplastic large cell lymphoma	0.000[e]	0.999
ALK- peripheral T-cell lymphoma	6.083 (2.227-16.617)	< 0.001	ALK- peripheral T-cell lymphoma	10.180 (3.287-31.531)	< 0.001
**International Prognostic Index**[f]			**Total metabolic tumor volume**[g]		
Low-intermediate risk	5.787 (1.146-29.217)	0.034	Second quartile	0.519 (0.044-6.088)	0.601
High-intermediate risk	9.668 (2.030-46.044)	0.004	Third quartile	4.170 (0.728-23.875)	0.109
High risk	8.396 (1.594-44.230)	0.012	Fourth quartile	8.638 (1.706-43.743)	0.009
**Loss of appetite**			**Loss of appetite**		
Continuous variable (per 1% increase)	1.015 (1.004-1.026)	0.007	Continuous variable (per 1% increase)	1.007 (0.995-1.020)	0.249

ALK+, anaplastic lymphoma kinase-positive; ALK-, ALK-negative
Nagelkerke's R^2 (top to bottom), left column 0.240 – 0.253 – 0.258, right column 0.251 – 0.259 – 0.255
[a] Odds ratio (95% confidence interval)
[b] Wald test
[c] Reference, diffuse large B-cell lymphoma (total metabolic tumor volume was only available for diffuse large B-cell lymphoma and T-cell lymphoma)
[d] Reference, ≤ 60 years
[e] No progression events at end of treatment
[f] Reference, low risk
[g] Reference, first quartile

Table S5. Association of pretreatment quality of life with long-term outcome

QLQ-C30 Domain	Freedom from progression at 5 years[a]			Progression-free survival at 5 years[a]			Overall survival at 5 years[a]		
	QLQ-C30 low[b]	QLQ-C30 high[b]	p[c]	QLQ-C30 low[b]	QLQ-C30 high[b]	p[c]	QLQ-C30 low[b]	QLQ-C30 high[b]	p[c]
**Global quality of life**	71.1 (65.6-76.6)	79.2 (74.3-84.1)	0.028	62.5 (56.8-68.2)	74.5 (69.2-79.8)	0.001	71.5 (66.2-76.8)	84.2 (79.9-88.5)	0.001
**Functioning**									
Physical	70.3 (65.2-75.4)	81.8 (76.7-86.9)	<0.001	62.4 (57.1-67.7)	76.9 (71.4-82.4)	< 0.001	72.8 (67.9-77.7)	84.7 (80.0-89.4)	0.001
Role	71.5 (66.4-76.6)	79.0 (73.7-84.3)	0.050	64.9 (59.6-70.2)	72.0 (66.1-77.9)	0.054	75.1 (70.2-80.0)	80.5 (75.4-85.6)	0.206
Emotional	71.3 (65.8-76.8)	78.6 (73.7-83.5)	0.007	64.2 (58.5-69.9)	72.0 (66.7-77.3)	0.014	74.4 (69.3-79.5)	80.7 (76.0-85.4)	0.109
Cognitive	71.5 (66.0-77.0)	78.4 (73.3-83.5)	0.016	63.8 (58.1-69.5)	72.8 (67.5-78.1)	0.001	73.0 (67.7-78.3)	82.5 (77.8-87.2)	0.002
Social	72.7 (67.6-77.8)	78.1 (72.6-83.6)	0.069	65.9 (60.6-71.2)	71.4 (65.5-77.3)	0.065	75.3 (70.6-80.0)	80.5 (75.4-85.6)	0.139
**Symptoms**									
Fatigue	79.5 (74.8-84.2)	69.8 (63.9-75.7)	0.010	73.6 (68.5-78.7)	62.1 (56.0-68.2)	0.003	81.4 (76.9-85.9)	73.2 (67.7-78.7)	0.024
Nausea and vomiting	76.3 (72.2-80.4)	70.9 (62.7-79.1)	0.556	69.7 (65.4-74.0)	63.8 (55.4-72.2)	0.524	79.7 (75.8-83.6)	71.0 (63.0-79.0)	0.047
Pain	75.6 (70.5-80.7)	74.5 (69.2-79.8)	0.846	68.4 (62.9-73.9)	68.2 (62.7-73.7)	0.804	78.0 (73.1-82.9)	77.4 (72.3-82.5)	0.846
Dyspnea	81.6 (76.9-86.3)	67.8 (61.9-73.7)	0.003	74.3 (69.2-79.4)	61.8 (55.9-67.7)	< 0.001	80.8 (76.1-85.5)	74.1 (68.8-79.4)	0.017
Insomnia	77.3 (72.8-81.8)	70.6 (64.1-77.1)	0.058	70.5 (65.6-75.4)	63.9 (57.2-70.6)	0.021	80.2 (75.9-84.5)	72.8 (66.5-79.1)	0.021
Loss of appetite	79.5 (75.0-84.0)	69.0 (62.9-75.1)	0.009	73.0 (68.1-77.9)	62.2 (55.9-68.5)	0.002	82.1 (77.8-86.4)	71.8 (66.1-77.5)	0.001
Constipation	75.1 (70.6-79.6)	74.6 (67.7-81.5)	0.483	68.8 (64.1-73.5)	66.5 (59.2-73.8)	0.262	78.8 (74.7-82.9)	73.7 (66.8-80.6)	0.239
Diarrhea	75.5 (71.2-79.8)	73.8 (66.2-81.4)	0.674	68.2 (63.7-72.7)	68.7 (60.7-76.7)	0.667	78.0 (73.9-82.1)	75.4 (68.0-82.8)	0.644
Financial difficulties	74.5 (69.8-79.2)	75.7 (69.4-82.0)	0.872	68.0 (63.1-72.9)	68.1 (61.4-74.8)	0.572	78.5 (74.2-92.8)	75.0 (68.7-81.3)	0.544

Note that higher scores denote better performance in the global-quality-of-life and functional scales, while higher scores represent worse performance in the symptom scales.
[a] Kaplan-Meier estimate of the percentage of patients not progressing and/or surviving after 5 years (95% confidence interval)
[b] Low, patients with scores below or equal to the median of all observed scores; high, patients with scores above the median
[c] Log-rank test

Table S6. Multivariable Cox regression analysis of baseline factors with potential impact on long-term outcome

**Variables**<br>**Lymphoma subtype, International Prognostic Index, quality of life**			**Variables**<br>**Age group, lymphoma subtype, total metabolic tumor volume, quality of life**		
**Variable level**	**Hazard ratio[a]**	**p[b]**	**Variable level**	**Hazard ratio[a]**	**p[b]**
**Freedom from progression**					
**Lymphoma subtype[c]**			**Age group[d]**		
Primary mediastinal B-cell lymphoma	0.403 (0.128-1.276)	0.122	> 60 years	1.047 (0.706-1.554)	0.820
Follicular lymphoma grade 3	0.878 (0.458-1.685)	0.696	**Lymphoma subtype[c]**		
ALK+ anaplastic large cell lymphoma	0.377 (0.052-2.741)	0.335	ALK+ anaplastic large cell lymphoma	0.000 (0.000->100)	0.959
ALK- peripheral T-cell lymphoma	3.499 (2.201-5.564)	< 0.001	ALK- peripheral T-cell lymphoma	4.837 (2.949-7.936)	< 0.001
**International Prognostic Index[e]**			**Total metabolic tumor volume[f]**		
Low-intermediate risk	1.908 (1.197-3.041)	0.007	Second quartile	1.078 (0.531-2.191)	0.835
High-intermediate risk	2.889 (1.809-4.613)	< 0.001	Third quartile	2.343 (1.241-4.425)	0.009
High risk	3.702 (2.244-6.105)	< 0.001	Fourth quartile	4.209 (2.311-7.664)	< 0.001
**Physical functioning[g]**			**Physical functioning[g]**		
Pretreatment score high	0.711 (0.495-1.021)	0.064	Pretreatment score high	0.735 (0.482-1.121)	0.153
**Lymphoma subtype[d]**			**Age group[c]**		
Primary mediastinal B-cell lymphoma	0.425 (0.134-1.344)	0.145	> 60 years	1.057 (0.712-1.568)	0.785
Follicular lymphoma grade 3	0.896 (0.466-1.724)	0.742	**Lymphoma subtype[d]**		
ALK+ anaplastic large cell lymphoma	0.417 (0.058-3.025)	0.387	ALK+ anaplastic large cell lymphoma	0.000 (0.000->100)	0.958
ALK- peripheral T-cell lymphoma	3.622 (2.283-5.746)	< 0.001	ALK- peripheral T-cell lymphoma	4.980 (3.040-8.158)	< 0.001
**International Prognostic Index[e]**			**Total metabolic tumor volume[f]**		
Low-intermediate risk	1.987 (1.249-3.163)	0.004	Second quartile	1.115 (0.550-2.263)	0.762
High-intermediate risk	3.000 (1.881-4.785)	< 0.001	Third quartile	2.399 (1.278-4.503)	0.006
High risk	4.026 (2.457-6.597)	< 0.001	Fourth quartile	4.376 (2.420-7.913)	< 0.001

**Cognitive functioning**[g]			**Cognitive functioning**[g]		
Pretreatment score high	0.778 (0.555-1.092)	0.147	Pretreatment score high	0.679 (0.458-1.007)	0.054
**Progression-free survival**					
**Lymphoma subtype**[c]			**Age group**[d]		
Primary mediastinal B-cell lymphoma	0.317 (0.117-0.858)	0.024	> 60 years	1.715 (1.242-2.367)	0.001
Follicular lymphoma grade 3	0.714 (0.405-1.257)	0.243	**Lymphoma subtype**[c]		
ALK+ anaplastic large cell lymphoma	0.202 (0.028-1.456)	0.112	ALK+ anaplastic large cell lymphoma	0.000 (0.000->100)	0.939
ALK- peripheral T-cell lymphoma	2.840 (1.884-4.281)	< 0.001	ALK- peripheral T-cell lymphoma	3.388 (2.203-5.211)	< 0.001
**International Prognostic Index**[e]			**Total metabolic tumor volume**[f]		
Low-intermediate risk	1.548 (1.065-2.249)	0.022	Second quartile	1.315 (0.797-2.169)	0.284
High-intermediate risk	2.232 (1.529-3,258)	< 0.001	Third quartile	1.595 (0.972-2.616)	0.065
High risk	3.364 (2.270-4.986)	< 0.001	Fourth quartile	3.053 (1.923-4.848)	< 0.001
**Physical functioning**[g]			**Physical functioning**[g]		
Pretreatment score high	0.655 (0.486-0.883)	0.005	Pretreatment score high	0.670 (0.478-0.939)	0.020
**Lymphoma subtype**[c]			**Age group**[d]		
Primary mediastinal B-cell lymphoma	0.329 (0.122-0.891)	0.029	> 60 years	1.747 (1.264-2.413)	< 0.001
Follicular lymphoma grade 3	0.723 (0.409-1.276)	0.263	**Lymphoma subtype**[c]		
ALK+ anaplastic large cell lymphoma	0.227 (0.032-1.635)	0.141	ALK+ anaplastic large cell lymphoma	0.000 (0.000->100)	0.959
ALK- peripheral T-cell lymphoma	2.984 (1.959-4.438)	< 0.001	ALK- peripheral T-cell lymphoma	3.487 (2.269-5.361)	< 0.001
**International Prognostic Index**[e]			**Total metabolic tumor volume**[f]		
Low-intermediate risk	1.630 (1.124-2.365)	0.010	Second quartile	1.392 (0.844-2.293)	0.195
High-intermediate risk	2.335 (1.601-3.406)	< 0.001	Third quartile	1.668 (1.021-2.725)	0.041
High risk	3.796 (2.577-5.592)	< 0.001	Fourth quartile	3.212 (2.034-5.073)	< 0.001

**Cognitive functioning**[g]			**Cognitive functioning**[g]		
Pretreatment score high	0.786 (0.596-1.036)	0.088	Pretreatment score high	0.653 (0.475-0.897)	0.009
**Overall survival**					
**Lymphoma subtype**[c]			**Age group**[d]		
Primary mediastinal B-cell lymphoma	0.100 (0.014-0.714)	0.022	> 60 years	2.541 (1.751-3.688)	< 0.001
Follicular lymphoma grade 3	0.448 (0.208-9,965)	0.040	**Lymphoma subtype**[c]		
ALK+ anaplastic large cell lymphoma	0.000 (0.000->100)	0.956	ALK+ anaplastic large cell lymphoma	0.000 (0.000->100)	0.947
ALK- peripheral T-cell lymphoma	2.836 (1.823-4.413)	< 0.001	ALK- peripheral T-cell lymphoma	3.198 (2.009-5.089)	< 0.001
**International Prognostic Index**[e]			**Total metabolic tumor volume**[f]		
Low-intermediate risk	1.320 (0.851-2.047)	0.215	Second quartile	1.208 (0.705-2.070)	0.492
High-intermediate risk	2.235 (1.466-3.408)	< 0.001	Third quartile	1.368 (0.800-2.341)	0.252
High risk	3.605 (2.346-5.539)	< 0.001	Fourth quartile	2.640 (1.620-4.302)	< 0,001
**Physical functioning**[g]			**Physical functioning**[g]		
Pretreatment score high	0,700 (0.502-0.978)	0.036	Pretreatment score high	0.692 (0.478-1.000)	0.050
**Lymphoma subtype**[d]			**Age group**[c]		
Primary mediastinal B-cell lymphoma	0.102 (0.014-0.731)	0.023	> 60 years	2.625 (1.805-3.816)	< 0.001
Follicular lymphoma grade 3	0.444 (0.205-0.906)	0.039	**Lymphoma subtype**[d]		
ALK+ anaplastic large cell lymphoma	0.000 (0.000->100)	0.956	ALK+ anaplastic large cell lymphoma	0.000 (0.000->100)	0.964
ALK- peripheral T-cell lymphoma	2.956 (1.902-4.592)	< 0.001	ALK- peripheral T-cell lymphoma	3.265 (2.052-5.196)	< 0.001
**International Prognostic Index**[e]			**Total metabolic tumor volume**[f]		
Low-intermediate risk	1.389 (0.898-2.150)	0.140	Second quartile	1.275 (0.745-2.183)	0.375
High-intermediate risk	2.272 (1.491-3.462)	< 0.001	Third quartile	1.414 (0.830-2.409)	0.203
High risk	3.955 (2.593-6.033)	< 0.001	Fourth quartile	2.725 (1.678-4.423)	< 0.001

**Cognitive functioning**[g]			**Cognitive functioning**[g]		
Pretreatment score high	0.730 (0.534-0.997)	0.048	Pretreatment score high	0.597 (0.419-0.850)	0.004

ALK+, anaplastic lymphoma kinase-positive; ALK-, ALK-negative
[a] Hazard ratio (95% confidence interval)
[b] Wald test
[c] Reference, diffuse large B-cell lymphoma
[d] Reference, ≤ 60 years
[e] Reference, low risk
[f] Reference, first quartile
[g] Reference, pretreatment score low (patients with scores below or equal to the median of all observed scores)

Table S7. Quality of life at three months of follow-up in PETAL trial participants in relation to the age- and gender-matched German reference population

QLQ-C30 Domain	Female			Male		
	Mean[a]			Mean[a]		
	PETAL trial	Reference population[b]	p[c]	PETAL trial	Reference population[b]	p[c]
**Global quality of life**	67.1 (±20.7)	64.8	0.251	72.6 (±17.9)	66.7	<0.001
**Functioning**						
Physical	80.3 (±18.3)	82.5	0.221	83.3 (±17.7)	86.5	0.022
Role	71.3 (±25.8)	77.0	0.025	73.3 (±25.9)	80.2	0.001
Emotional	76.0 (±19.5)	67.4	<0.001	77.0 (±22.1)	72.9	0.021
Cognitive	81.7 (±22.0)	83.6	0.390	80.3 (±22.1)	83.8	0.047
Social	75.4 (±24.9)	80.8	0.028	78.9 (±24.7)	82.6	0.062
**Symptoms**						
Fatigue	29.3 (±22.6)	32.9	0.102	29.4 (±23.5)	28.5	0.623
Nausea and vomiting	4.0 (±10.7)	4.7	0.486	2.2 (±7.3)	3.2	0.080
Pain	18.7 (±25.5)	31.5	<0.001	14.9 (±22.9)	26.0	<0.001
Dyspnea	18.7 (±22.6)	18.0	0.741	16.1 (±24.8)	16.7	0.778
Insomnia	30.4 (±32.2)	33.9	0.277	22.4 (±27.9)	25.0	0.248
Loss of appetite	10.6 (±22.4)	8.5	0.347	6.5 (±16.1)	7.4	0.482
Constipation	7.9 (±18.7)	8.7	0.646	9.1 (±21.5)	5.9	0.066
Diarrhea	7.3 (±19.6)	9.4	0.275	8.0 (±20.3)	9.4	0.395
Financial difficulties	19.4 (±30.2)	14.3	0.089	18.8 (±29.7)	13.2	0.020
**Number of assessments**	105	2,634		160	2,050	

Note that higher scores denote better performance in the global-quality-of-life and functional scales, while higher scores represent worse performance in the symptom scales.
[a] Mean (± standard deviation for the PETAL trial population; standard deviations were not available for the reference population)
[b] Waldmann et al. *PLoS One*. 2013;8(9):e74149
[c] One-sample t-test

Table S8. Quality-of-life changes during treatment and follow-up of 82 patients with complete longitudinal quality-of-life data

QLQ-C30 Domain	Baseline		Interim PET		End of treatment		3-month follow-up		6-month follow-up		9-month follow-up		12-month follow-up	
	**Median[a]**	**Mean[b]**	**Mean[b]**	**p[c]**	**Mean[b]**	**p[c]**	**Mean[b]**	**p[c]**	**Mean[b]**	**p[c]**	**Mean[b]**	**p[c]**	**Mean[b]**	**p[c]**
**Global quality of life**	66.7 (50.0-83.3)	61.1 (±23.3)	59.5 (±18.9)	0.582	63.4 (±17.9)	0.436	73.8 (±16.5)	< 0.001	71.7 (±20.4)	0.004	71.3 (±18.5)	0.002	73.6 (±17.4)	< 0.001
**Functioning**														
Physical	86.7 (78.8-100)	81.6 (±19.5)	71.9 (±20.3)	< 0.001	72.9 (±21.3)	0.001	83.6 (±15.3)	0.375	84.6 (±15.0)	0.166	82.4 (±15.4)	0.734	84.5 (±14.6)	0.186
Role	83.3 (50.0-100)	73.3 (±29.8)	61.0 (±31 1)	0.002	60.5 (±28.0)	0.003	75.6 (±23.6)	0.549	75.4 (±24.5)	0.405	74.2 (±25.8)	0.712	75.8 (±25.1)	0.428
Emotional	66.7 (41.7-91.7)	65.6 (±24.5)	73.3 (±22.7)	< 0.001	74.0 (±20.9)	0.003	79.5 (±17.9)	< 0.001	74.9 (±20.7)	0.006	77.9 (±19.3)	< 0.001	78.6 (±19.0)	< 0.001
Cognitive	100 (66.7-100)	84.6 (±23.5)	85.2 (±19.6)	0.853	82.1 (±24.7)	0.344	80.1 (±23.5)	0.114	80.5 (±23.4)	0.167	80.9 (±23.7)	0.201	80.2 (±23.1)	0.139
Social	66.7 (50.0-100)	67.1 (±32.2)	66.9 (±26.7)	0.883	74.4 (±23.9)	0.055	78.9 (±24.0)	0.003	80.5 (±23.4)	< 0.001	80.1 (±23.2)	< 0.001	83.7 (±22.4)	< 0.001
**Symptoms**														
Fatigue	33.3 (22.2-44.4)	35.4 (±25.0)	43.2 (±22.6)	0.004	41.2 (±23.7)	0.063	27.6 (±17.9)	0.004	29.5 (±23.1)	0.074	30.2 (±22.6)	0.105	28.8 (±22.8)	0.053
Nausea and vomiting	0.0 (0.0-0.0)	9.6 (±23.3)	7.5 (±12.9)	0.421	5.3 (±13.3)	0.113	3.7 (±10.5)	0.032	4.1 (±11.6)	0.045	3.7 (±9.8)	0.027	2.8 (±10.7)	0.020
Pain	16.7 (0.0-50.0)	27.8 (±31.7)	21.3 (±26.9)	0.092	13.8 (±21.2)	< 0.001	15.2 (±21.8)	< 0.001	15.9 (±24.1)	0.001	16.1 (±22.7)	0.002	13.0 (±21.3)	< 0.001
Dyspnea	0.0 (0.0-33.3)	19.2 (±29.4)	23.9 (±29.0)	0.141	24.8 (±28.6)	0.145	16.3 (±21.8)	0.373	18.5 (±24.2)	0.902	18.7 (±22.3)	0.822	19.1 (±24.0)	1.000
Insomnia	33.3 (0.0-66.7)	36.6 (±33.2)	32.1 (±30.9)	0.200	30.9 (±32.2)	0.193	22.8 (±28.1)	< 0.001	27.6 (±29.7)	0.033	25.6 (±30.7)	0.010	28.5 (±29.2)	0.051
Loss of appetite	0.0 (0.0-66.7)	26.8 (±36.5)	22.0 (±31.1)	0.246	18.7 (±31.0)	0.043	5.8 (±17.3)	< 0.001	6.6 (±17.0)	< 0.001	4.9 (±16.7)	< 0.001	6.1 (±14.0)	< 0.001
Constipation	0.0 (0.0-33.3)	16.9 (±32.4)	23.0 (±33.2)	0.114	12.8 (±26.1)	0.302	8.2 (±20.8)	0.017	10.7 (±21.0)	0.104	11.0 (±22.3)	0.150	9.9 (±21.4)	0.055
Diarrhea	0.0 (0.0-0.0)	8.1 (±23.4)	6.7 (±14.3)	0.677	11.9 (±24.2)	0.199	7.0 (±20.1)	0.791	8.2 (±17.8)	1.000	5.3 (±15.2)	0.334	7.5 (±18.2)	0.880
Financial difficulties	0.0 (0.0-33.3)	16.5 (±25.0)	22.4 (±32.3)	0.055	23.9 (±29.5)	0.031	19.1 (±28.2)	0.348	16.7 (±26.3)	0.909	17.1 (±27.8)	0.804	12.8(±23.3)	0.288

Note that higher scores denote better performance in the global-quality-of-life and functional scales, while higher scores represent worse performance in the symptom scales.
[a] Median (interquartile range [quartile 1 - quartile 3])
[b] Mean (± standard deviation)
[c] Compared to baseline assessment (paired samples t-test)

Table S9. Quality of life during treatment and follow-up of interim PET-negative patients with CD20-positive lymphoma allocated to 6 cycles of R-CHOP or 6 cycles of R-CHOP followed by two doses of rituximab (R)

QLQ-C30 Domain	Baseline					Interim PET			End of treatment		
	Median[a]		Mean[b]			Mean[b]			Mean[b]		
	6xR-CHOP	6xR-CHOP + 2xR	6xR-CHOP	6xR-CHOP + 2xR	p[c]	6xR-CHOP	6xR-CHOP + 2xR	p[c]	6xR-CHOP	6xR-CHOP + 2xR	p[c]
**Global quality of life**	50.0 (33.3-70.8)	62.5 (41.7-75.0)	54.1 (+24.1)	58.7 (+24.2)	0.059	57.5 (+20.9)	59.5 (+21.1)	0.418	60.8 (+22.0)	61.9 (+17.0)	0.635
**Functioning**											
Physical	86.7 (60.0-100)	86.7 (73.3-100)	77.0 (+24.3)	79.9 (+23.7)	0.230	68.6 (+24.2)	71.1 (+21.4)	0.336	69.9 (+23.1)	71.9 (+19.8)	0.424
Role	66.7 (33.3-100)	66.7 (50.0-100)	62.4 (+35.9)	68.0 (+34.0)	0.112	54.2 (+32.4)	57.3 (+30.4)	0.387	56.4 (+31.2)	56.9 (+27.4)	0.893
Emotional	58.3 (41.7-83.3)	66.7 (41.7-83.3)	59.7 (+27.3)	62.6 (+26.7)	0.277	66.8 (+26.3)	67.9 (+23.0)	0.699	70.3 (+22.7)	68.3 (+22.2)	0.458
Cognitive	100 (66.7-100)	100 (66.7-100)	82.5 (+23.3)	83.2 (+21.9)	0.748	81.8 (+23.2)	82.0 (+21.5)	0.936	81.6 (+24.5)	78.9 (+23.6)	0.353
Social	66.7 (37.5-100)	66.7 (50.0-100)	64.4 (+31.9)	67.9 (+30.5)	0.265	63.9 (+29.8)	64.1 (+28.1)	0.942	67.5 (+30.3)	65.6 (+27.0)	0.596
**Symptoms**											
Fatigue	33.3 (22.2-66.7)	33.3 (11.1-55.6)	39.8 (+29.3)	36.7 (+28.4)	0.290	48.5 (+27.5)	47.0 (+25.5)	0.606	44.5 (+27.6)	44.0 (+25.8)	0.871
Nausea and vomiting	0.0 (0.0-0.0)	0.0 (0.0-0.0)	6.4 (+17.0)	7.4 (+19.0)	0.595	9.8 (+17.8)	9.5 (+15.7)	0.867	8.1 (+17.6)	4.1 (+12.7)	0.031
Pain	16.7 (0.0-66.7)	16.7 (0.0-50.0)	32.5 (+34.3)	28.8 (+33.1)	0.279	24.6 (+29.1)	21.2 (+26.9)	0.288	16.3 (+24.1)	20.8 (+27.1)	0.153
Dyspnea	0.0 (0.0-33.3)	0.0 (0.0-33.3)	25.7 (+33.4)	24.7 (+32.1)	0.771	25.0 (+31.0)	27.2 (+30.8)	0.545	28.9 (+30.9)	27.7 (+29.2)	0.751
Insomnia	33.3 (0.0-66.7)	33.3 (0.0-66.7)	35.3 (+34.2)	39.4 (+35.0)	0.247	36.0 (+32.3)	37.3 (+33.4)	0.739	29.7 (+33.1)	39.5 (+35.8)	0.021
Loss of appetite	0.0 (0.0-33.3)	0.0 (0.0-33.3)	24.7 (+33.9)	21.0 (+30.1)	0.251	25.8 (+32.4)	22.0 (+32.2)	0.313	23.0 (+31.8)	15.3 (+29.2)	0.039
Constipation	0.0 (0.0-33.3)	0.0 (0.0-33.3)	17.1 (+29.5)	15.5 (+28.7)	0.584	24.3 (+33.9)	18.7 (+28.8)	0.125	13.8 (+26.6)	12.8 (+23.4)	0.736
Diarrhea	0.0 (0.0-0.0)	0.0 (0.0-0.0)	10.9 (+24.0)	10.4 (+22.1)	0.828	11.9 (+23.8)	11.2 (+22.8)	0.805	13.8 (+25.7)	10.6 (+20.5)	0.255
Financial difficulties	0.0 (0.0-33.3)	0.0 (0.0-33.3)	19.4 (+29.4)	16.1 (+28.5)	0.265	23.5 (+32.2)	25.1 (+32.6)	0.674	25.6 (+33.6)	28.0 (+30.9)	0.547
**Number of assessments**	224	174	224	174		176	126		166	113	

Table S9 continued.

QLQ-C30 Domain	3-month follow-up			6-month follow-up			9-month follow-up			12-month follow-up		
	Mean[b]			Mean[b]			Mean[b]			Mean[b]		
	6xR-CHOP	6xR-CHOP + 2xR	p[c]	6xR-CHOP	6xR-CHOP + 2xR	p[c]	6xR-CHOP	6xR-CHOP + 2xR	p[c]	6xR-CHOP	6xR-CHOP + 2xR	p[c]
**Global quality of life**	72.2 (+17.9)	68.8 (+18.4)	0.153	72.0 (+20.4)	70.5 (+20.1)	0.606	72.8 (+18.4)	67.2 (+23.0)	0.065	73.3 (+19.3)	73.5 (+16.5)	0.935
**Functioning**												
Physical	83.4 (+18.0)	82.0 (+16.1)	0.518	83.2 (+18.3)	82.4 (+15.8)	0.733	83.6 (+16.7)	80.8 (+16.9)	0.244	85.4 (+17.2)	83.4 (+15.4)	0.418
Role	74.2 (+23.2)	72.3 (+27.0)	0.580	77.1 (+25.7)	71.1 (+25.0)	0.089	76.4 (+74.6)	74.5 (+26.5)	0.615	79.5 (+23.0)	74.4 (+25.5)	0.176
Emotional	79.5 (+19.5)	73.9 (+21.0)	0.039	77.1 (+21.6)	71.1 (+23.2)	0.062	78.8 (+19.3)	70.2 (+23.7)	0.006	77.9 (+21.2)	72.8 (+22.4)	0.129
Cognitive	83.3 (+20.4)	77.8 (+22.7)	0.057	82.2 (+23.2)	76.9 (+23.8)	0.109	81.3 (+21.7)	75.1 (+24.2)	0.058	84.8 (+19.0)	77.3 (+25.4)	0.033
Social	78.8 (+25.4)	77.0 (+23.3)	0.570	81.3 (+24.1)	76.9 (+25.1)	0.210	82.5 (+22.5)	76.0 (+26.7)	0.069	84 6 (+20.6)	80.6 (+21.4)	0.215
**Symptoms**												
Fatigue	26.3 (+21.3)	31.3 (+24.1)	0.100	27.6 (+22.1)	32.3 (+23.7)	0.144	27.8 (+21.9)	35.4 (+23.9)	0.021	27.0 (+22.1)	31.3 (+23.3)	0.222
Nausea and vomiting	2.5 (+7.8)	3.2 (+9.7)	0.554	3.2 (+10.4)	3.1 (+10.3)	0.974	2.4 (+8.0)	6.1 (+14.4)	0.031	3.0 (+9.7)	4.2 (+13.4)	0.509
Pain	14.8 (+23.2)	16.2 (+22.3)	0.652	15.4 (+25.0)	18.9 (+26.5)	0.337	15.4 (+22.9)	20.2 (+26.4)	0.171	13.5 (+22.2)	16.9 (+23.8)	0.333
Dyspnea	17.7 (+25.4)	16.3 (+21.4)	0.666	14.2 (+22.7)	22.1 (+28.0)	0.030	15.7 (+22.1)	22.2 (+26.9)	0.063	15.6 (+22.2)	27.5 (+26.7)	0.002
Insomnia	23.4 (+29.2)	27.3 (+29.0)	0.305	22.8 (+27.7)	34.8 (+33.1)	0.006	22.2 (+30.0)	33.7 (+32.4)	0.010	24.1 (+28.7)	34.2 (+30.5)	0.026
Loss of appetite	9.6 (+20.3)	6.1 (+16.7)	0.143	7.2 (+17.9)	7.4 (+19.8)	0.944	6.3 (+18.6)	11.5 (+24.0)	0.094	7.7 (+20.4)	9.5 (+17.9)	0.541
Constipation	8.1 (+20.1)	9.1 (+21.2)	0.732	6.9 (+16.7)	10.3 (+23.5)	0.249	9.5 (+21.0)	12.7 (+24.4)	0.316	9.0 (+19.8)	9.5 (+21.7)	0.879
Diarrhea	6.8 (+17.2)	8.0 (+21.3)	0.640	7.3 (+17.9)	6.5 (+17.5)	0.753	8.7 (+20.7)	5.6 (+14.4)	0.211	5.9 (+15.7)	4.1 (+12.3)	0.392
Financial difficulties	20.8 (+31.7)	16.3 (+27.4)	0.251	18.8 (+314)	18.8 (+28.2)	0.994	18.8 (+30.0)	20.1 (+28.4)	0.765	14.2 (+23.2)	15.3 (+26.0)	0.777
**Number of assessments**	135	100		120	90		117	90		105	75	

Note that higher scores denote better performance in the global-quality-of-life and functional scales, while higher scores represent worse performance in the symptom scales.
[a] Median (interquartile range [quartile 1 - quartile 3])
[b] Mean (± standard deviation)
[c] Welch test

Table S10. Quality of life during treatment and follow-up of interim PET-positive patients randomly assigned to eight cycles of R-CHOP or two cycles of R-CHOP followed by six cycles of the Burkitt protocol

	Baseline					Interim PET			Before cycle 5			End of treatment		
	Median[a]		Mean[b]			Mean[b]			Mean[b]			Mean[b]		
QLQ-C30 Domain	8xR-CHOP	2xR-CHOP + 6xBurkitt pr.	8xR-CHOP	2xR-CHOP + 6xB. pr.	p[c]	8xR-CHOP	2xR-CHOP + 6xB-Prot.	p[c]	8xR-CHOP	2xR-CHOP + 6xB. pr.	p[c]	8xR-CHOP	2xR-CHOP + 6xB. pr.	p[c]
**Global quality of life**	50.0 (39.6-66.7)	62.5 (33.3-83.3)	51.7 (±24.2)	53.5 (±30.9)	0.805	50.9 (±24.3)	54.9 (±24.9)	0.575	55.8 (±30.7)	61.7 (±27.4)	0.718	44.6 (±28.4)	59.5 (±22.1)	0.135
**Functioning**														
Physical	86.7 (51.7-95.0)	80.0 (26.7-100)	74.4 (±27.4)	68.5 (±35.5)	0.493	65.2 (±22.8)	64.8 (±25.6)	0.953	62.0 (±32.2)	65.3 (±24.2)	0.827	50.0 (±32.4)	59.6 (±27.2)	0.402
Role	66.7 (33.3-100)	66.7 (29.2-100)	56.9 (±38.2)	62.2 (±38.2)	0.611	54.5 (±34.8)	46.2 (±29.1)	0.374	41.7 (±30.7)	46.7 (±27.4)	0.756	35.7 (±31.9)	44.0 (±25.8)	0.455
Emotional	58.3 (33.3-83.3)	62.5 (31.3-83.3)	61.4 (±28.1)	57.9 (±29.2)	0.652	66.4 (±23.7)	64.8 (±21.8)	0.809	69.2 (±31.7)	63.3 (±29.2)	0.732	60.1 (±29.6)	55.6 (±30.5)	0.691
Cognitive	91.7 (66.7-100)	83.3 (66.7-100)	82.2 (±21.9)	79.5 (±26.4)	0.677	85.8 (±23.4)	76.5 (±23.9)	0.180	90.0 (±17.9)	80.0 (±18.3)	0.344	83.3 (±19.6)	65.5 (±22.1)	0.033
Social	50.0 (25.0-83.3)	58.3 (16.7-100)	54.0 (±33.2)	55.8 (±38.6)	0.859	61.7 (±28.0)	63.6 (±30.7)	0.823	63.3 (±29.2)	40.0 (±34.6)	0.236	51.3 (±24.0)	41.7 (±40.2)	0.455
**Symptoms**														
Fatigue	33.3 (11.1-69.4)	33.3 (8.3-80.6)	41.9 (±31.9)	41.9 (±36.8)	0.998	49.0 (±26.6)	48.0 (±27.3)	0.899	64.4 (±23.9)	55.6 (±28.3)	0.566	65.9 (±23.3)	54.0 (±32.6)	0.277
Nausea and vomiting	0.0 (0.0-16.7)	0.0 (0.0-16.7)	11.7 (±23.6)	10.3 (±20.0)	0.810	12.3 (±22.5)	6.8 (±11.1)	0.269	10.0 (±17.9)	6.7 (±9.1)	0.641	21.4 (±31.0)	13.1 (±27.1)	0.455
Pain	33.3 (0.0-66.7)	33.3 (0.0-70.8)	37.8 (±36.3)	37.2 (±38.4)	0.953	28.4 (±30.2)	33.3 (±34.5)	0.601	20.0 (±32.2)	33.3 (±40.8)	0.545	39.3 (±27.4)	40.5 (±37.4)	0.924
Dyspnea	0.0 (0.0-33.3)	0.0 (0.0-66.7)	17.8 (±24.3)	26.9 (±38.9)	0.306	25.9 (±32.5)	27.3 (±28.4)	0.878	20.0 (±35.8)	40.0 (±36.5)	0.344	31.0 (±33.2)	47.6 (±36.3)	0.217
Insomnia	16.7 (0.0-41.7)	33.3 (0.0-66.7)	27.8 (±34.0)	35.9 (±36.4)	0.395	30.9 (±30.6)	31.8 (±31.7)	0.916	33.3 (±35.1)	26.7 (±27.9)	0.698	42,9 (±44.2)	40.5 (±39.6)	0.882
Loss of appetite	0.0 (0.0-66.7)	33.3 (0.0-66.7)	24.4 (±33.8)	34.6 (±35.9)	0.282	30.9 (±35.7)	21.2 (±26.3)	0.282	30.0 (±42.9)	33.3 (±47.1)	0.898	38.1 (±36.6)	28.6 (±28.8)	0.452
Constipation	0.0 (0.0-8.3)	0.0 (0.0-41.7)	14.4 (±29.9)	23.1 (±36.2)	0.340	16.0 (±25.1)	18.2 (±28.6)	0.785	20.0 (±28.1)	6.7 (±14.9)	0.252	19.0 (±33.9)	9.5 (±15.6)	0.352
Diarrhea	0.0 (0.0-33.3)	0.0 (0.0-33.3)	12.2 (±22.3)	17.9 (±28.6)	0.413	9.9 (±22.3)	10.6 (±18.9)	0.902	6.7 (±14.1)	33.3 (±47.1)	0.278	14.3 (±28.4)	19.0 (±38.6)	0.713
Financial difficulties	0.0 (0.0-33.3)	0.0 (0.0-50.0)	20.0 (±27.1)	24.0 (±31.2)	0.618	23.5 (±27.4)	22.7 (±26.0)	0.925	26.7 (±34.4)	13.3 (±18.3)	0.345	33 3 (±39.2)	28.6 (±31.6)	0.727
**Number of assessments**	30	26	30	26		27	22		10	5		14	14	

Table S10 continued.

QLQ-C30 Domain	3-month follow-up			6-month follow-up			9-month follow-up			12-month follow-up		
	Mean[b]			Mean[b]			Mean[b]			Mean[b]		
	8xR-CHOP	2xR-CHOP + 6xB. pr.	p[c]	8xR-CHOP	2xR-CHOP + 6xB. pr.	p[c]	8xR-CHOP	2xR-CHOP + 6xB. pr.	p[c]	8xR-CHOP	2xR-CHOP + 6xB. pr.	p[c]
**Global quality of life**	51.9 (±33.8)	81.5 (±10.8)	0.032	75.0 (±19.2)	81.9 (±27.1)	0.613	70.4 (±20.5)	75.0 (±18.0)	0.695	51.7 (±27.3)	72.9 (±15.8)	0.190
**Functioning**												
Physical	68.9 (±20.5)	87.4 (±15.1)	0.046	83.8 (±18.4)	90.0 (±16.7)	0.538	73.3 (±22.9)	80.0 (±14.9)	0.523	64.7 (±21.8)	81.7 (±15.8)	0.217
Role	53.7 (±37.1)	75.9 (±14.7)	0.124	78.6 (±31.5)	83.3 (±27.9)	0.778	59.3 (±32.4)	63.3 (±29.8)	0.818	36.7 (±32.1)	75.0 (±21.5)	0.071
Emotional	68.5 (±31.9)	78.7 (±18.2)	0.421	76.2 (±19.5)	88.9 (±14.6)	0.208	71.3 (±25.7)	63.3 (±34.7)	0.668	67.8 (±30.5)	79.2 (±10.8)	0.472
Cognitive	79.6 (±24,7)	81.5 (±13.0)	0.846	88.1 (±20.9)	86.1 (±19.5)	0.863	75.9 (±30.2)	60.0 (±30.3)	0.372	73.3 (±27.9)	75.0 (±9.6)	0.906
Social	63.0 (±35.1)	66.7 (±26.4)	0.804	78.6 (±18.5)	88.9 (±13.6)	0.274	75.9 (±23.7)	70.0 (±29.8)	0.714	66.7 (±33.3)	70.8 (±21.0)	0.826
**Symptoms**												
Fatigue	40.7 (±25.5)	22.2 (±14.7)	0.082	24.6 (±12.8)	14.8 (±24.0)	0.399	28.4 (±27.8)	37.8 (±29.0)	0.572	44.4 (±34.2)	16.7 (±19.2)	0.172
Nausea and vomiting	9.3 (±14.7)	0.0 (±0.0)	0.714	0.0 (±0.0)	2.8 (±6.8)	1.000	1.9 (±5.6)	6.7 (±14.9)	0.520	6.7 (±14.9)	0.0 (±0.0)	1.000
Pain	37.0 (±38.9)	22.2 (±20.4)	0.331	21.4 (±20.9)	5.6 (±13.6)	0.130	16.7 (±23.6)	20.0 (±27.4)	0.825	20.0 (±21.7)	20.8 (±21.0)	0.955
Dyspnea	29.6 (±26.1)	18.5 (±29.4)	0.409	14.3 (±26.2)	11.1 (±17.2)	0.799	22.2 (±28.9)	20.0 (±18.3)	0.863	6.7 (±14.9)	33.3 (±0.0)	0.016
Insomnia	48.1 (±47.5)	14.8 (±24.2)	0.085	38.1 (±35.6)	11.1 (±17.2)	0.110	44.4 (±33.3)	26.7 (±27.9)	0.313	20.0 (±29.8)	33.3 (±0.0)	0.374
Loss of appetite	20.8 (±30.5)	0.0 (±0.0)	0.786	0.0 (±0.0)	0.0 (±0.0)	1.000	3.7 (±11.1)	6.7 (±14.9)	0.710	13.3 (±18.3)	8.3 (±16.7)	0.681
Constipation	11.1 (±23.6)	3.7 (±11.1)	0.411	9.5 (±16.3)	0.0 (±0.0)	0.786	3.7 (±11.1)	0.0 (±0.0)	1.000	20.0 (±18.3)	0.0 (±0.0)	0.533
Diarrhea	14.8 (±33.8)	3.7 (±11.1)	0.371	19.0 (±26.2)	0.0 (±0.0)	0.786	14.8 (±33.8)	13.3 (±29.8)	0.934	13.3 (±29.8)	0.0 (±0.0)	0.800
Financial difficulties	29.6 (±35.1)	7.4 (±14.7)	0.109	9.5 (±16.3)	5.6 (±13.6)	0.641	18.5 (±29.4)	40.0 (±43.5)	0.361	26.7 (±27.9)	8.3 (±16.7)	0.263
**Number of assessments**	9	9		7	6		9	5		5	4	

Note that higher scores denote better performance in the global-quality-of-life and functional scales, while higher scores represent worse performance in the symptom scales.
B. pr., Burkitt protocol
[a] Median (interquartile range [quartile 1 - quartile 3])
[b] Mean (± standard deviation)
[c] Welch test (substituted by Mann-Whitney U test in case of 0 variance)

Table S11. Quality of life at the end of treatment in interim PET-negative patients versus interim PET-positive patients

QLQ-C30 Domain	End-of-treatment assessment					
	Median[a]			Mean[b]		
	iPET negative	iPET positive	p[c]	iPET negative	iPET positive	p[d]
**Global quality of life**	66.7 (50.0-75.0)	50.0 (33.3-83.3)	0.086	61.5 (±20.4)	52.1 (±26.1)	0.074
**Functioning**						
Physical	73.3 (53.3-86.7)	60.0 (35.0-80.0)	0.006	71.0 (±22.0)	54.8 (±29.8)	0.009
Role	66.7 (33.3-83.3)	41.7 (4.2-66.7)	0.004	57.3 (+29.7)	39.9 (±28.8)	0.004
Emotional	75.0 (50.0-91.7)	62.5 (33.3-81.3)	0.040	69.9 (±22.6)	57.8 (±29.6)	0.044
Cognitive	83.3 (66.7-100)	83.3 (50.0-100)	0.067	80.8 (±24.0)	74.4 (±22.4)	0.160
Social	66.7 (50.0-100)	50.0 (16.7-66.7)	0.002	67.1 (±29.3)	46.3 (±33.1)	0.004
**Symptoms**						
Fatigue	33.3 (33.3-66.7)	61.1 (36.1-86.1)	0.003	43.7 (±26.7)	59.9 (±28.4)	0.007
Nausea and vomiting	0.0 (0.0-0.0)	0.0 (0.0-16.7)	0.003	6.3 (±15.5)	17.3 (±28.6)	0.056
Pain	0.0 (0.0-33.3)	33.3 (16.7-66.7)	< 0.001	17.4 (±25.2)	39.9 (±32.2)	0.001
Dyspnea	33.3 (0.0-33.3)	33.3 (0.0-66.7)	0.123	28.1 (±29.9)	39.3 (±35.2)	0.113
Insomnia	33.3 (0.0-66.7)	33.3 (33.3-91.7)	0.317	33.1 (±34.1)	41.7 (±41.2)	0.295
Loss of appetite	0.0 (0.0-33.3)	33.3 (0.0-66.7)	0.010	19.1 (±30.4)	33.3 (±32.7)	0.034
Constipation	0.0 (0.0-33.3)	0.0 (0.0-33.3)	0.745	13.5 (±25.7)	14.3 (±26.3)	0.883
Diarrhea	0.0 (0.0-33.3)	0.0 (0.0-25.0)	0.794	11.9 (±23.2)	16.7 (±33.3)	0.464
Financial difficulties	0.0 (0.0-33.3)	33.3 (0.0-66.7)	0.501	26.0 (32.1)	31.0 (±35.1)	0.478
**Number of assessments**	299[e]	28[f]		299[e]	28[f]	

Note that higher scores denote better performance in the global-quality-of-life and functional scales, while higher scores represent worse performance in the symptom scales. iPET, interim PET

[a] Median (interquartile range [quartile 1 - quartile 3])
[b] Mean (± standard deviation)
[c] Mann-Whitney U test
[d] Welch test
[e] 279 patients treated with rituximab and 20 patients treated without rituximab
[f] 23 patients treated with rituximab and 5 patients treated without rituximab

Table S12. Quality of life at the end of treatment in relation to interim PET response and remission status

QLQ-C30 Domain	Complete remission[a]			Partial remission[a]			Stable disease[a]			Progressive disease[a]		
	iPET negative	iPET positive	p[b]	iPET negative	iPET positive	p[b]	iPET negative	iPET positive	p[b]	iPET negative	iPET positive	p[b]
**Global quality of life**	60.7 (±20.4)	58.3 (±24.3)	0.709	64.6 (±19.3)	54.2 (±30.8)	0.547	66.7 (±28.9)	70.8 (±17.7)	0.854	41.7 (±35.4)	26.7 (±19.0)	0.656
**Functioning**												
Physical	71.2 (±21.6)	63.3 (±24.7)	0.233	70.4 (±23.3)	65.0 (±40.1)	0.808	75.6 (±25.2)	63.3 (±4.7)	0.493	60.0 (±28.3)	23.0 (±22.1)	0.276
Role	57.1 (±29.5)	43.8 (±27.8)	0.083	58.2 (±30.4)	29.2 (±21.0)	0.063	66.7 (±33.3)	66.7 (±23.6)	1.000	33.3 (±47.1)	33.3 (±35.4)	1.000
Emotional	68.2 (±23.7)	57.5 (±32.7)	0.214	75.0 (±18.6)	58.3 (±32.6)	0.383	66.7 (±22.0)	62.5 (±5.9)	0.781	62.5 (±17.7)	53.3 (±31.5)	0.654
Cognitive	79.6 (±24.2)	75.0 (±19.2)	0.376	84.2 (±22.6)	70.8 (±25.0)	0.365	66.7 (±44.1)	83.3 (±23.6)	0.622	100 (±0.0)	70.0 (±36.1)	0.381
Social	67.0 (±29.4)	49.0 (±34.7)	0.059	68.0 (±28.9)	66.7 (±23.6)	0.918	66.7 (±33.3)	41.7 (±35.4)	0.506	33.3 (±23.6)	20.8 (±31.5)	0.626
**Symptoms**												
Fatigue	44.0 (±26.9)	48.6 (±27.2)	0.522	42.8 (±25.7)	66.7 (±27.2)	0.177	33.3 (±33.3)	55.6 (±15.7)	0.393	66.7 (±47.1)	88.9 (±19.2)	0.624
Nausea and vomiting	5.6 (±14.3)	6.3 (±10.3)	0.811	8.3 (±19.0)	16.7 (±23.6)	0.534	11.1 (±9.6)	8.3 (±11.8)	0.809	0.0 (±0.0)	60.0 (±43.5)	0.190
Pain	19.1 (±25.6)	35.4 (±26.4)	0.029	11.9 (±22.0)	54.2 (±25.0)	0.040	5.6 (±9.6)	0.0 (±0.0)	0.800	58.3 (±58.9)	63.3 (±44.7)	0.927
Dyspnea	30.4 (±30.1)	41.7 (±33.3)	0.208	22.1 (±28.8)	16.7 (±33.3)	0.770	11.1 (±19.2)	16.7 (±23.6)	0.809	33.3 (±47.1)	60.0 (±43.5)	0.570
Insomnia	34.7 (±34.5)	37.5 (±36.3)	0.769	28.4 (±32.5)	33.3 (±47.1)	0.848	22.2 (±19.2)	16.7 (±23.6)	0.809	50.0 (±70.7)	60.0 (±54.8)	0.878
Loss of appetite	18.2 (±29.3)	25.0 (±25.8)	0.327	20.7 (±33.0)	50.0 (±57.7)	0.386	22.2 (±19.2)	33.3 (±47.1)	0.797	50.0 (±70.7)	40.0 (±27.9)	0.875
Constipation	14.4 (±26.0)	8.3 (±19.2)	0.248	11.0 (±24.9)	16.7 (±33.3)	0.757	0.0 (±0.0)	16.7 (±23.6)	0.400	33.3 (±47.1)	33.3 (±40.8)	1.000
Diarrhea	12.4 (±24.3)	14.6 (±29.7)	0.776	10.9 (±20.7)	8.3 (±16.7)	0.781	0.0 (±0.0)	0.0 (±0.0)	1.000	16.7 (±23.6)	40.0 (±54.8)	0.469
Financial difficulties	24.4 (±31.2)	25.0 (±25.8)	0.929	31.1 (±34.6)	25.0 (±31.9)	0.734	22.2 (±19.2)	33.3 (±47.1)	0.797	0.0 (±0.0)	60.0 (±54.8)	0.381
**Number of assessments**	219	16		74	4		3	2		2	5	

Note that higher scores denote better performance in the global-quality-of-life and functional scales, while higher scores represent worse performance in the symptom scales.
iPET, interim PET
The analysis was restricted to 325 patients because the remission status of two patients was not reported.
[a] Mean (± standard deviation)
[b] Welch test (substituted by Mann-Whitney U test in case of 0 variance)

Table S13. Diffuse large B-cell lymphoma subgroup - Association of pretreatment quality of life with patient baseline features

QLQ-C30 Domain	All patients		Age groups[b]			Gender[b]			B Symptoms[b]		
	Median[a]	Mean[b]	≤ 60 years	> 60 years	p[c]	Female	Male	p[c]	Absent	Present	p[c]
**Global quality of life**	50.0 (33.3-75.0)	55.0 (±25.2)	57.7 (±24.6)	52.4 (±25.6)	0.037	51.0 (±25.4)	57.7 (±24.7)	0.010	60.3 (±22.7)	42.4 (±26.2)	< 0.001
**Functioning**											
Physical	86.7 (60.0-100)	76.3 (±25.9)	78.5 (±24.7)	74.1 (±26.8)	0.086	70.9 (±27.0)	79.9 (±24.4)	< 0.001	81.3 (±22.6)	64.1 (±28.9)	< 0.001
Role	66.7 (33.3-100)	63.4 (±36.9)	61.9 (±36.7)	64.7 (±37.1)	0.456	56.5 (±37.3)	68.1 (±35.9)	0.002	70.5 (±33.7)	46.4 (±38.6)	< 0.001
Emotional	66.7 (41.7-83.3)	61.3 (±27.9)	59.0 (±28.0)	63.5 (±27.8)	0.107	54.6 (±27.2)	65.8 (±27.5)	< 0.001	65.1 (±26.7)	52.3 (±28.9)	< 0.001
Cognitive	83.3 (66.7-100)	81.7 (±23.8)	82.8 (±22.8)	80.7 (±24.7)	0.375	80.2 (±24.8)	82.8 (±23.1)	0.302	85.5 (±20.9)	72.6 (±27.6)	< 0.001
Social	66.7 (33.3-100)	65.0 (±32.3)	61.1 (±31.9)	68.7 (±32.3)	0.018	60.7 (±34.1)	67.9 (±30.8)	0.031	69.5 (±30.0)	53.9 (±35.0)	< 0.001
**Symptoms**											
Fatigue	33.3 (11.1-66.7)	39.4 (±29.9)	39.1 (±29.4)	39.7 (±30.5)	0.843	46.2 (±30.3)	34.7 (±28.8)	< 0.001	32.5 (±27.0)	55.6 (±30.3)	< 0.001
Nausea and vomiting	0.0 (0.0-0.0)	8.0 (±19.6)	7.8 (±18.8)	8.3 (±20.4)	0.827	11.7 (±23.8)	5..5 (±15.8)	0.004	4.3 (±14.8)	16.9 (±25.9)	< 0.001
Pain	25.0 (0.0-66.7)	33.8 (±35.0)	35.5 (±35.3)	32.2 (±34.6)	0.338	36.3 (±35.3)	32.1 (±34.7)	0.244	30.4 (±33.9)	42.3 (±36.2)	0.002
Dyspnea	0.0 (0.0-33.3)	24.4 (±32.8)	24.4 (±32.8)	24.5 (±32.8)	0.972	29.6 (±34.9)	20.8 (±30.8)	0.010	18.3 (±28.7)	38.7 (±37.2)	< 0.001
Insomnia	33.3 (0.0-66.7)	38.3 (±35.3)	41.3 (±35.9)	35.3 (±34.7)	0.092	41.3 (±35.9)	36.3 (±34.9)	0.169	33.6 (±33.0)	49.3 (±38.3)	< 0.001
Loss of appetite	0.0 (0.0-66.7)	26.9 (±34.4)	26.4 (±32.8)	27.5 (±35.9)	0.751	35.2 (±37.0)	21.3 (±31.3)	< 0.001	18.1 (±28.9)	47.9 (±37.2)	< 0.001
Constipation	0.0 (0.0-33.3)	17.2 (±29.9)	15.4 (±28.9)	19.0 (±30.8)	0.230	16.4 (±29.5)	17.8 (±30.2)	0.637	13.8 (±26.2)	25.6 (±36.1)	0.002
Diarrhea	0.0 (0.0-0.0)	10.9 (±23.5)	12.4 (±24.4)	9.4 (±22.6)	0.201	12.5 (±25.6)	9.8 (±22.0)	0.274	9.9 (±22.8)	13.3 (±25.2)	0.213
Financial difficulties	0.0 (0.0-33.3)	18.0 (±28.8)	22.9 (±31.3)	13.4 (±25.3)	0.001	17.6 (±27.6)	18.3 (±29.6)	0.813	16.2 (±27.7)	22.5 (±30.9)	0.057
**Number of assessments**	400	400	196	204		162	238		280	119	

Table S13 continued.

QLQ-C30 Domain	International Prognostic Index[b]					Total metabolic tumor volume[bd]				
	Low	Low-intermediate	High-intermediate	High	p[c]	1st quartile	2nd quartile	3rd quartile	4th quartile	p[c]
**Global quality of life**	64.7 (±19.7)	55.7 (±24.5)	49.4 (±26.2)	35.7 (±25.2)	< 0.001	62.6 (±22.5)	62.0 (±21.5)	48.1 (±24.0)	42.3 (±25.8)	< 0.001
**Functioning**										
Physical	86.8 (±15.3)	78.0 (±22.9)	71.8 (±29.5)	51.3 (±29.6)	< 0.001	89.3 (±14.3)	82.4 (±21.4)	72.9 (±28.2)	59.4 (±29.6)	< 0.001
Role	78.0 (±26.5)	62.2 (±36.9)	55.6 (±39.1)	37.8 (±40.3)	< 0.001	83.1 (±24.1)	71.0 (±32.5)	53.7 (±37.6)	42.4 (±40.1)	< 0.001
Emotional	66.3 (±25.4)	63.2 (±26.8)	53.4 (±30.5)	54.9 (±29.9)	0.003	65.6 (±27.2)	61.7 (±27.5)	61.8 (±25.8)	51.5 (±31.0)	0.018
Cognitive	86.7 (±19.0)	83.3 (±24.5)	76.5 (±25.3)	72.6 (±28.3)	< 0.001	87.9 (±19.7)	84.1 (±23.0)	82.3 (±22.7)	73.2 (±28.6)	0.003
Social	71.5 (±26.6)	63.3 (±32.1)	62.1 (±36.7)	54.5 (±37.4)	0.005	71.3 (±27.4)	70.7 (±30.4)	60.6 (±32.5)	52.5 (±37.2)	< 0.001
**Symptoms**										
Fatigue	27.4 (±23.0)	40.3 (±29.0)	46.6 (±32.2)	59.6 (±30.7)	< 0.001	24.2 (±20.3)	34.0 (±29.1)	43.5 (±28.9)	57.4 (±31.4)	< 0.001
Nausea and vomiting	4.4 (±10.6)	8.3 (±20.8)	8.5 (±16.9)	19.2 (±31.4)	< 0.001	2.5 (±6.5)	4.3 (±10.3)	11.2 (±22.4)	16.5 (±28.8)	< 0.001
Pain	24.7 (±29.5)	37.1 (±35.5)	36.5 (±37.1)	48.3 (±38.5)	< 0.001	18.8 (±24.2)	31.2 (±35.7)	40.4 (±35.7)	44.7 (±39.5)	< 0.001
Dyspnea	14.4 (±25.8)	23.8 (±30.6)	34.6 (±37.8)	38.5 (±37.4)	< 0.001	13.3 (±24.1)	14.9 (±26.7)	27.6 (±33.5)	43.3 (±37.2)	< 0.001
Insomnia	31.0 (±31.7)	38.1 (±37.9)	47.4 (±36.2)	46.1 (±35.3)	0.002	33.3 (±35.4)	34.5 (±32.7)	40.7 (±34.2)	51.4 (±38.7)	0.008
Loss of appetite	16.8 (±26.6)	24.8 (±32.4)	34.2 (±36.4)	47.8 (±41.8)	< 0.001	14.2 (±23.6)	21.2 (±30.8)	33.7 (±36.1)	42.4 (±40.0)	< 0.001
Constipation	10.6 (±21.1)	17.1 (±29.3)	24.2 (±37.7)	25.4 (±35.2)	0.001	13.5 (±24.8)	13.3 (±25.5)	15.9 (±29.7)	26.7 (±37.0)	0.035
Diarrhea	9.4 (±21.0)	11.2 (±23.8)	9.6 (±21.0)	15.8 (±31.2)	0.508	7.5 (±17.6)	8.8 (±20.9)	9.2 (±22 3)	15.3 (±27.0)	0.175
Financial difficulties	16.2 (±26.4)	21.8 (±31.1)	19.9 (±32.1)	13.5 (±25.1)	0.242	15.2 (±25.7)	17.9 (±30.6)	16.0 (±25.9)	23.4 (±33.4)	0.321
**Number of assessments**	157	105	78	60		79	81	78	84	

Note that higher scores denote better performance in the global-quality-of-life and functional scales, while higher scores represent worse performance in the symptom scales.
[a] Median (interquartile range [quartile 1 - quartile 3])
[b] Mean (± standard deviation)
[c] Welch test
[d] First quartile, 0.2-39.9 cm^3; second quartile, 40-228.9 cm^3; third quartile, 229-720.9 cm^3; fourth quartile, 721-5937 cm^3

Table S14. Diffuse large B-cell lymphoma subgroup - Association of pretreatment quality of life with interim and end-of-treatment response

QLQ-C30 Domain	Interim PET response[a]			End-of treatment response[a]				
	Favorable	Unfavorable	p[b]	Complete remission	Partial remission	Stable disease	Progressive disease	p[b]
**Global quality of life**	55.2 (±24.9)	53.8 (±27.4)	0.749	57.8 (±23.8)	52.1 (±25.8)	60.8 (±24.9)	40.4 (±29.5)	0.046
**Functioning**								
Physical	76.7 (±24.8)	72.9 (±33.5)	0.476	80.8 (±21.9)	71.3 (±28.4)	71.3 (±32.4)	55.0 (±35.9)	0.003
Role	64.0 (±36.1)	58.3 (±43.1)	0.408	68.7 (±34.0)	55.4 (±39.5)	60.0 (±37.8)	39.2 (±45.0)	0.007
Emotional	61.4 (±27.8)	60.6 (±29.3)	0.869	62.4 (±27.7)	59.7 (±27.3)	65.0 (±28.5)	55.0 (±31.3)	0.656
Cognitive	82.1 (±23.6)	79.2 (±25.7)	0.480	83.1 (±21.8)	82.0 (±26.2)	80.0 (±30.2)	74.2 (±28.3)	0.587
Social	65.8 (±31.7)	58.1 (±36.4)	0.192	66.9 (±30.7)	63.7 (±33.3)	61.7 (±37.7)	50.0 (±39.7)	0.308
**Symptoms**								
Fatigue	39.4 (±29.3)	38.9 (±35.1)	0.924	35.4 (±28.0)	42.3 (±30.3)	46.7 (±35.1)	61.7 (±35.8)	0.012
Nausea and vomiting	7.8 (±19.6)	9.8 (±19.8)	0.523	6.4 (±16.6)	9.3 (±22.1)	15.0 (±31.9)	17.5 (±23.9)	0.161
Pain	33.6 (±34.8)	36.0 (±36.5)	0.678	32.7 (±34.1)	35.4 (±35.3)	18.3 (±21.4)	41.7 (±39.9)	0.145
Dyspnea	24.6 (±32.2)	22.7 (±37.2)	0.748	20.8 (±30.4)	27.4 (±33.0)	40.0 (±41.0)	43.3 (±42.0)	0.048
Insomnia	39.3 (±35.3)	30.3 (±35.1)	0.115	35.4 (±34.7)	41.6 (±36.7)	50.0 (±28.3)	45.0 (±37.9)	0.227
Loss of appetite	26.7 (±34.1)	28.8 (±37.1)	0.722	23.7 (±33.0)	26.3 (±33.3)	26.7 (±34.4)	56.7 (±39.1)	0.012
Constipation	16.8 (±29.6)	20.5 (±32.3)	0.480	16.9 (±29.9)	13.9 (±28.3)	10.0 (±22.5)	28.3 (32.9±)	0.275
Diarrhea	10.7 (±23.6)	12.1 (±22.8)	0.705	11.1 (±23.8)	10.1 (±23.7)	10.0 (±22.5)	16.7 (±27.6)	0.811
Financial difficulties	17.8 (±28.9)	20.2 (±28.3)	0.607	16.9 (±28.6)	23.2 (±30.7)	14.8 (±24.2)	10.5 (±27.3)	0.252
**Number of assessments**	356	44		262	90	10	20	

Note that higher scores denote better performance in the global-quality-of-life and functional scales, while higher scores represent worse performance in the symptom scales.
[a] Mean (± standard deviation)
[b] Welch test

Table S15. Diffuse large B-cell lymphoma subgroup - Multivariable Cox regression analysis of baseline factors with potential impact on long-term outcome

**Variables**<br>**Lymphoma subtype, International Prognostic Index, quality of life**			**Variables**<br>**Age group, lymphoma subtype, total metabolic tumor volume, quality of life**		
**Variable level**	**Hazard ratio[a]**	**p[b]**	**Variable level**	**Hazard ratio[a]**	**p[b]**
**Freedom from progression**					
**International Prognostic Index[c]**			**Age group[d]**		
Low-intermediate risk	2.210 (1.266-3.858)	0.005	> 60 years	1.007 (0.648-1.564)	0.976
High-intermediate risk	3.259 (1.868-5.684)	< 0.001	**Total metabolic tumor volume[e]**		
High risk	4.439 (2.465-7.994)	< 0.001	Second quartile	1.174 (0.485-2.839)	0.722
**Physical functioning[f]**			Third quartile	2.898 (1.345-6.248)	0.007
Pretreatment score high	0.712 (0.468-1.082)	0.111	Fourth quartile	5.242 (2.473-11.110)	< 0.001
			**Physical functioning[f]**		
			Pretreatment score high	0.776 (0.485-1.243)	0.292
**International Prognostic Index[c]**			**Age group[d]**		
Low-intermediate risk	2.306 (1.325-4.016)	0.003	> 60 years	1.009 (0.650-1.566)	0.969
High-intermediate risk	3.252 (1.859-5.686)	< 0.001	**Total metabolic tumor volume[e]**		
High risk	4.992 (2.804-8.885)	< 0.001	Second quartile	1.1226 (0.508-2.959)	0.651
**Cognitive functioning[f]**			Third quartile	3.014 (1.408-6.448)	0.004
Pretreatment score high	0.822 (0.556-1.215)	0.325	Fourth quartile	5.442 (2.601-11.381)	< 0.001
			**Cognitive functioning[f]**		
			Pretreatment score high	0.788 (0.507-1.227)	0.292

**Progression-free survival**					
**International Prognostic Index**[c]			**Age group**[d]		
Low-intermediate risk	1.674 (1.092-2.568)	0.018	> 60 years	1.747 (1.230-2.482)	0.002
High-intermediate risk	2.436 (1.585-3.744)	< 0.001	**Total metabolic tumor volume**[e]		
High risk	3.741 (2.395-5.842)	< 0.001	Second quartile	1.389 (0.784-2.460)	0.259
**Physical functioning**[f]			Third quartile	1.741 (1.000-3.028)	0.050
Pretreatment score high	0.665 (0.475-0.931)	0.017	Fourth quartile	3.361 (1.963-5.754)	< 0.001
			**Physical functioning**[f]		
			Pretreatment score high	0.690 (0.478-0.998)	0.048
**International Prognostic Index**[c]			**Age group**[d]		
Low-intermediate risk	1.765 (1.153-2.701)	0.009	> 60 years	1.780 (1.254-2.528)	0.001
High-intermediate risk	2.439 (1.585-3.755)	< 0.001	**Total metabolic tumor volume**[e]		
High risk	4.348 (2.810-6.727)	< 0.001	Second quartile	1.474 (0.834-2.607)	0.182
**Cognitive functioning**[f]			Third quartile	1.830 (1.057-3.168)	0.031
Pretreatment score high	0.813 (0.596-1.110)	0.193	Fourth quartile	3.503 (2.063-5.948)	< 0.001
			**Cognitive functioning**[f]		
			Pretreatment score high	0.702 (0.495-0.996)	0.047
**Overall survival**					
**International Prognostic Index**[c]			**Age group**[d]		
Low-intermediate risk	1.725 (1.061-2.802)	0.028	> 60 years	2.630 (1.751-3.952)	< 0.001
High-intermediate risk	2.467 (1.521-4.003)	< 0.001	**Total metabolic tumor volume**[e]		
High risk	4.207 (2.581-6.857)	< 0.001	Second quartile	1.185 (0.644-2.180)	0.585

**Physical functioning**[f]			Third quartile	1.365 (0.754-2.472)	0.304
Pretreatment score high	0.728 (0.502-1.056)	0.094	Fourth quartile	2.825 (1.603-4.979)	< 0.001
			**Physical functioning**[f]		
			Pretreatment score high	0.735 (0.490-1.101)	0.135
**International Prognostic Index**[c]			**Age group**[d]		
Low-intermediate risk	1.799 (1.110-2.917)	0.017	> 60 years	2.721 (1.810-4.091)	< 0.001
High-intermediate risk	2.437 (1.499-3.963)	< 0.001	**Total metabolic tumor volume**[e]		
High risk	4.728 (2.935-7.615)	< 0.001	Second quartile	1.1261 (0.687-2.316)	0.454
**Cognitive functioning**[f]			Third quartile	1.415 (0.785-2.548)	0.248
Pretreatment score high	0.813 (0.576-1.148)	0.239	Fourth quartile	2.860 (1.638-4.996)	< 0.001
			**Cognitive functioning**[f]		
			Pretreatment score high	0.665 (0.451-0.982)	0.040

Abbreviations: ALK+, anaplastic lymphoma kinase-positive; ALK-, ALK-negative
[a] Hazard ratio (95% confidence interval)
[b] Wald test
[c] Reference, low risk
[d] Reference, ≤ 60 years
[e] Reference, first quartile
[f] Reference, pretreatment score low (patients with scores below or equal to the median of all observed scores)

Table S16. Diffuse large B-cell lymphoma subgroup - Quality-of-life changes during treatment and follow-up

QLQ-C30 Domain	Baseline		Interim PET		End of treatment		3-month follow-up		6-month follow-up		9-month follow-up		12-month follow-up	
	Median[a]	Mean[b]	Mean[b]	p[c]	Mean[b]	p[c]	Mean[b]	p[c]	Mean[b]	p[c]	Mean[b]	p[c]	Mean[b]	p[c]
**Global quality of life**	58.3 (41.7-75.0)	56.6 (±24.4)	58.2 (±21.5)	0.383	60.7 (±20.8)	0.030	70.9 (±18.9)	< 0.001	71.8 (±20.3)	< 0.001	69.7 (±21.7)	< 0.001	72.7 (±19.3)	< 0.001
**Functioning**														
Physical	86.7 (66.7-100)	77.8 (±25.2)	69.5 (±23.0)	< 0.001	69.4 (±23.3)	< 0.001	83.0 (±17.0)	0.005	82.7 (±17.4)	0.011	81.5 (±17.5)	0.055	83.8 (±16.9)	0.003
Role	66.7 (33.3-100)	65.0 (±36.3)	55.5 (±31.8)	< 0.001	55.8 (±30.5)	< 0.001	75.2 (±25.2)	< 0.001	74.9 (±26.0)	< 0.001	74.1 (±27.2)	0.002	76.0 (±25.4)	< 0.001
Emotional	66.7 (41.7-83.3)	62.3 (±27.6)	68.1 (±25.2)	0.007	69.4 (±23.5)	< 0.001	78.3 (±19.2)	< 0.001	74.2 (±22.3)	< 0.001	74.3 (±22.7)	< 0.001	75.9 (±22.7)	< 0.001
Cognitive	100 (66.7-100)	83.0 (±23.2)	82.5 (±22.8)	0.754	79.8 (±24.8)	0.111	80.5 (±21.2)	0.194	79.4 (±23.7)	0.095	78.7 (±23.3)	0.049	81.6 (±22.2)	0.516
Social	66.7 (50.0-100)	65.8 (±31.5)	64.8 (±29.3)	0.670	65.4 (±29.9)	0.852	77.9 (±24.3)	< 0.001	78.9 (±24.9)	< 0.001	78.6 (±24.7)	< 0.001	82.6 (±21.3)	< 0.001
**Symptoms**														
Fatigue	33.3 (11.1-55.6)	37.6 (±29.1)	46.6 (±26.8)	< 0.001	45.3 (±27.5)	0.001	27.4 (±22.3)	< 0.001	29.1 (±22.4)	< 0.001	31.0 (±23.8)	0.006	28.4 (±23.4)	< 0.001
Nausea and vomiting	0.0 (0.0-0.0)	7.4 (±19.0)	9.2 (±17.2)	0.230	7.6 (±17.7)	0.883	2.7 (±8.2)	< 0.001	3.0 (±9.8)	< 0.001	4.7 (±12.1)	0.050	3.4 (±11.3)	0.004
Pain	16.7 (0.0-66.7)	32.7 (±34.7)	23.5 (±28.7)	< 0.001	19.2 (±25.8)	< 0.001	14.5 (±22.3)	< 0.001	17.1 (±25.4)	< 0.001	18.1 (±24.8)	< 0.001	16.2 (±23.7)	< 0.001
Dyspnea	0.0 (0.0-33.3)	21.8 (±30.7)	24.6 (±30.1)	0.258	27.9 (±30.1)	0.017	16.2 (±22.9)	0.016	17.7 (±25.3)	0.104	19.8 (±25.1)	0.426	20.3 (±24.4)	0.564
Insomnia	33.3 (0.0-66.7)	35.9 (±34.4)	35.6 (±33.1)	0.918	34.2 (±35.0)	0.558	24.8 (±28.7)	< 0.001	28.7 (±30.5)	0.015	27.9 (±31.3)	0.010	29.6 (±30.1)	0.046
Loss of appetite	0.0 (0.0-33.3)	24.3 (±33.1)	24.1 (±32.4)	0.932	20.7 (±30.8)	0.175	7.5 (±17.9)	< 0.001	7.2 (±19.1)	< 0.001	9.2 (±22.5)	< 0.001	8.9 (±19.8)	< 0.001
Constipation	0.0 (0.0-33.3)	16.3 (±29.4)	20.2 (±30.4)	0.111	13.7 (±25.4)	0.255	7.7 (±19.7)	< 0.001	8.1 (±19.9)	< 0.001	9.9 (±21.5)	0.006	8.6 (±20.4)	0.001
Diarrhea	0.0 (0.0-0.0)	10.9 (±23.8)	11.0 (±22.2)	0.956	12.1 (±23.2)	0.543	6.8 (±18.4)	0.028	6.8 (±17.9)	0.028	7.1 (±17.6)	0.043	7.2 (±17.8)	0.064
Financial difficulties	0.0 (0.0-33.3)	17.6 (±28.8)	23.7 (±31.6)	0.016	27.1 (±32.5)	< 0.001	17.4 (±29.0)	0.941	17.4 (±28.9)	0.944	19.7 (±29.5)	0.451	13.6 (±23.5)	0.115
**Number of assessments**	344		267		241		201		180		167		144	

Note that higher scores denote better performance in the global-quality-of-life and functional scales, while higher scores represent worse performance in the symptom scales.
[a] Median (interquartile range [quartile 1 - quartile 3])
[b] Mean (± standard deviation)
[c] Compared to baseline assessment (Welch test)

Table S17. Diffuse large B-cell lymphoma subgroup - Quality of life at the end of treatment in interim PET-negative patients versus interim-PET-positive patients

QLQ-C30 Domain	End-of-treatment assessment					
	Median[a]			Mean[b]		
	iPET negative	iPET positive	p[c]	iPET negative	iPET positive	p[d]
**Global quality of life**	66.7 (50.0-75.0)	58.3 (33.3-83.3)	0.759	60.9 (±20.3)	56.8 (±26.7)	0.550
**Functioning**						
Physical	73.3 (53.3-86.7)	60.0 (11.3-85.0)	0.045	70.6 (±22.0)	52.6 (±33.9)	0.053
Role	66.7 (33.3-83.3)	41.7 (0.0-66.7)	0.019	57.1 (±30.3)	37.5 (±29.5)	0.020
Emotional	75.0 (50.0-91.7)	66.7 (33.3-83.3)	0.257	70.1 (±22.9)	60.1 (±31.1)	0.225
Cognitive	83.3 (66.7-100)	83.3 (50.0-100)	0.221	80.2 (±24.8)	74.0 (±25.1)	0.347
Social	66.7 (50.0-100)	33.3 (16.7-66.7)	0.003	67.0 (±29.1)	42.7 (±32.8)	0.010
**Symptoms**						
Fatigue	33.3 (33.3-66.7)	61.1 (44.4-77.8)	0.039	44.4 (±27.2)	58.3 (±29.1)	0.081
Nausea and vomiting	0.0 (0.0-0.0)	0.0 (0.0-33.3)	0.016	6.8 (±16.3)	19.8 (±29.9)	0.105
Pain	0.0 (0.0-33.3)	33.3 (16.7-62.5)	0.007	18.0 (±24.9)	36.5 (±32.3)	0.040
Dyspnea	33.3 (0.0-33.3)	16.7 (0.0-58.3)	0.804	28.0 (±30.0)	27.1 (±32.7)	0.917
Insomnia	33.3 (0.0-66.7)	33.3 (0.0-66.7)	0.884	33.9 (±34.7)	37.5 (±40.1)	0.733
Loss of appetite	0.0 (0.0-33.3)	33.3 (0.0-66.7)	0.008	19.5 (±30.5)	37.5 (±31.9)	0.043
Constipation	0.0 (0.0-33.3)	0.0 (0.0-25.0)	0.935	13.8 (±25.6)	12.5 (±24.0)	0.835
Diarrhea	0.0 (0.0-33.3)	0.0 (0.0-0.0)	0.562	12.2 (±22.7)	12.5 (±29.5)	0.973
Financial difficulties	0.0 (0.0-33.3)	33.3 (0.0-66.7)	0.161	26.3 (±32.3)	37.5 (±36.3)	0.245
**Number of assessments**	225	16		225	16	

Note that higher scores denote better performance in the global-quality-of-life and functional scales, while higher scores represent worse performance in the symptom scales. iPET, interim PET
[a] Median (interquartile range [quartile 1 - quartile 3])
[b] Mean (± standard deviation)
[c] Mann-Whitney U test
[d] Welch test

Table S18. Diffuse large B-cell lymphoma subgroup - Quality of life at the end of treatment in relation to the remission status

QLQ-C30 Domain	End-of-treatment assessment[a]				
	Complete remission	Partial remission	Stable disease	Progressive disease	p[b]
**Global quality of life**	59.6 (±20.7)	65.7 (±19.0)	75.0 (±14.4)	33.3 (±20.4)	0.046
**Functioning**					
Physical	70.5 (±22.0)	70.4 (±23.4)	71.1 (±13.9)	28.3 (±32.2)	0.157
Role	55.6 (±30.4)	59.5 (±29.5)	66.7 (±16.7)	23.3 (±32.5)	0.206
Emotional	67.5 (±24.7)	76.6 (±18.1)	66.7 (±8.3)	50.0 (±30.6)	0.080
Cognitive	79.1 (±25.0)	82.5 (±23.9)	83.3 (±16.7)	70.0 (±36.1)	0.769
Social	66.1 (±29.6)	68.6 (±28.0)	50.0 (±28.9)	16.7 (±20.4)	0.011
**Symptoms**					
Fatigue	45.1 (±27.5)	42.2 (±25.7)	48.1 (±17.0)	84.4 (±29.0)	0.118
Nausea and vomiting	6.3 (±15.4)	8.8 (±18.9)	11.1 (±9.6)	40.0 (±43.5)	0.379
Pain	21.0 (±25.4)	10.7 (±20.0)	0.0 (±0.0)	70.0 (±41.5)	<0.001
Dyspnea	29.7 (±29.8)	22.0 (±29.4)	11.1 (±19.2)	46.7 (±44.7)	0.258
Insomnia	36.2 (±34.7)	25.4 (±32.4)	22.2 (±19.2)	60.0 (±54.8)	0.214
Loss of appetite	19.6 (±29.5)	19.8 (±32.8)	33.3 (±33.3)	53.3 (±38.0)	0.376
Constipation	13.8 (±25.0)	13.2 (±27.2)	11.1 (±19.2)	20.0 (±29.8)	0.967
Diarrhea	12.2 (±23.4)	12.0 (±21.2)	0.0 (±0.0)	26.7 (±43.5)	0.606
Financial difficulties	25.7 (±31.8)	29.9 (±33.2)	33.3 (±33.3)	40.0 (±54.8)	0.813
**Number of assessments**	173	59	3	5	

Note that higher scores denote better performance in the global-quality-of-life and functional scales, while higher scores represent worse performance in the symptom scales. The analysis was restricted to 240 (instead of 241) patients because the remission status of one patient was not reported.
[a] Mean (± standard deviation)
[b]† Welch test (substituted by Mann-Whitney U test in case of 0 variance)

2. Supplemental Figures

Figure S1

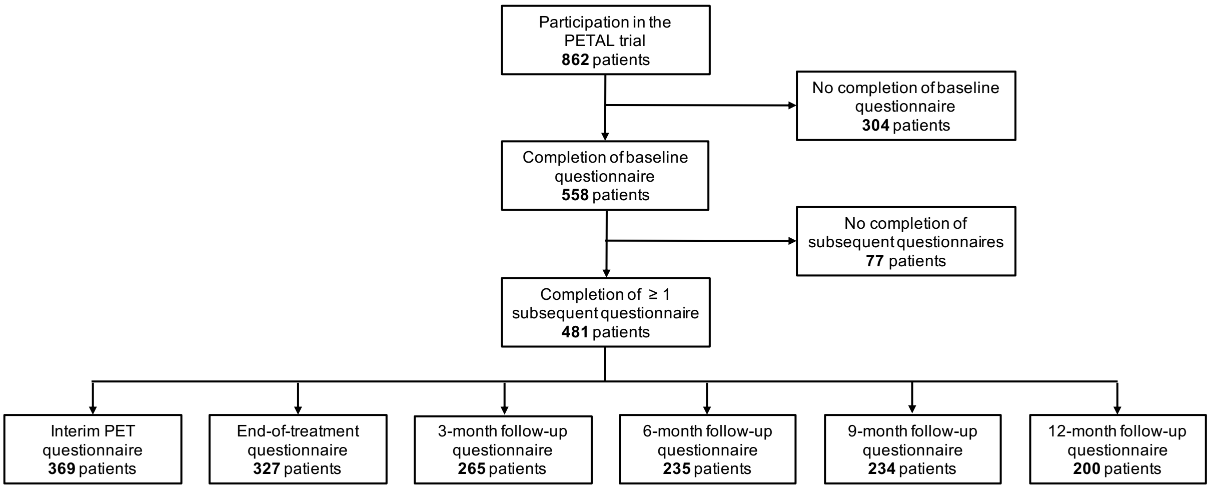

Flow-chart of patients completing the health-related quality of life questionnaires in the course of the PETAL trial

Reasons for ignoring the questionnaires at the indicated times were not provided by the trial sites.

Figure S2

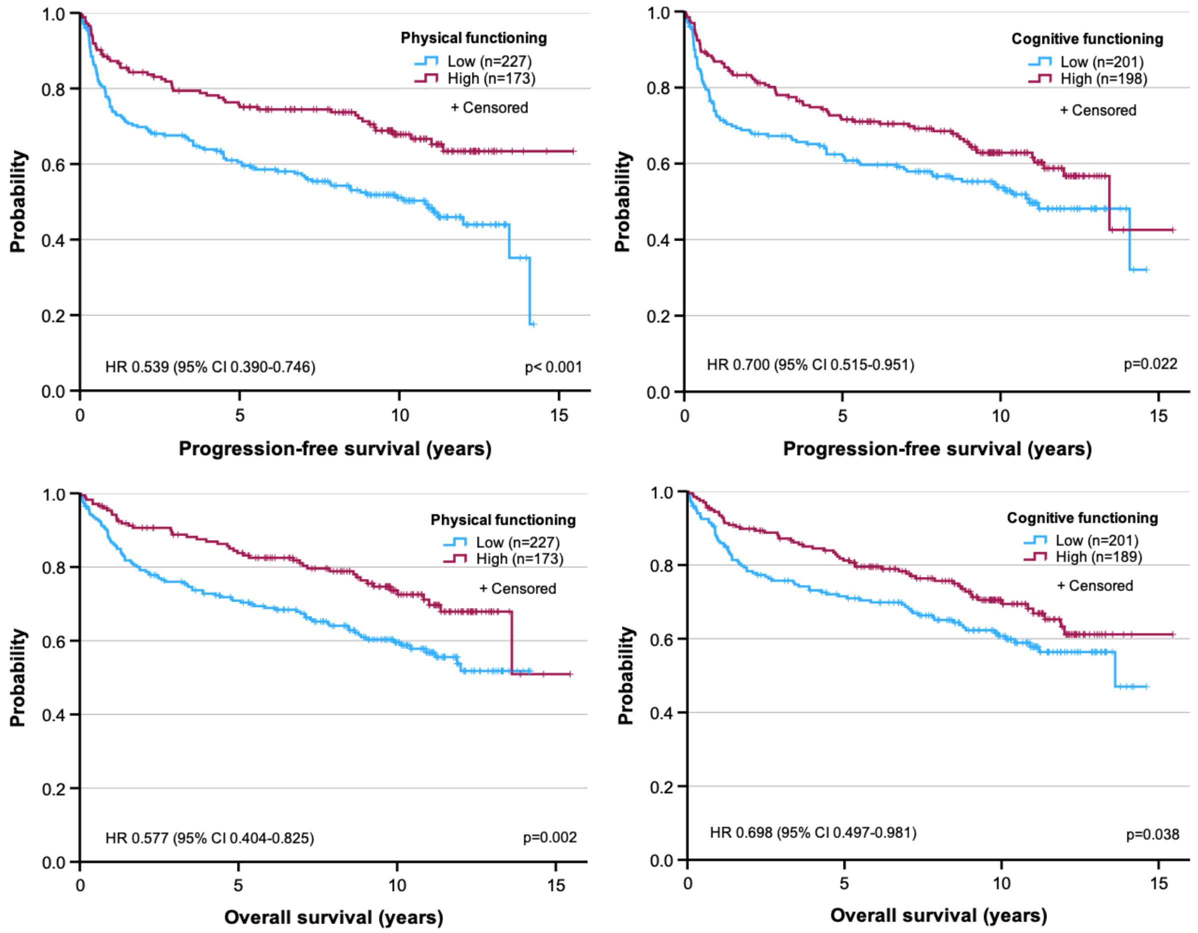

Diffuse large B-cell lymphoma subgroup - Progression-free survival (top) and overall survival (bottom) in relation to physical (left) or cognitive functioning (right) at the pretreatment quality-of-life assessment.

Low, patients with scores below or equal to the median of all observed scores; high, patients with scores above the median. HR, hazard ratio; CI, confidence interval; p, log-rank test

Figure S3

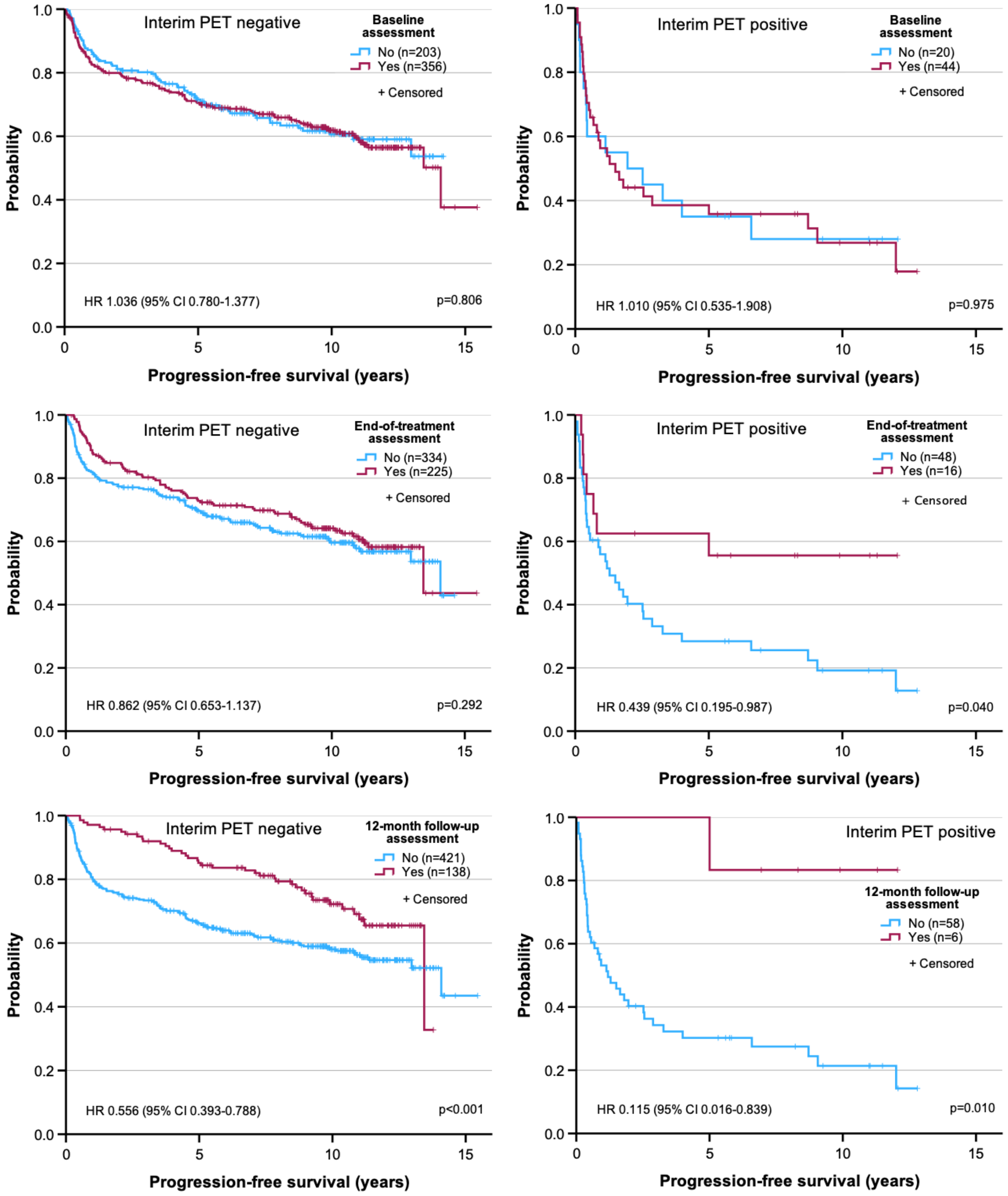

Diffuse large B-cell lymphoma subgroup - Progression-free survival in relation to completion versus non-completion of the quality-of-life questionnaire before treatment (top), at end of treatment (middle) and after 12 months of follow-up (bottom).

Left, interim PET negative patients (good prognosis); right, interim PET positive patients (poor prognosis). HR, hazard ratio; CI, confidence interval; p, log-rank test

3. Full list of sites, principal investigators, biostatisticians, reference pathologists, and members of the Data and Safety Monitoring Board

Sites and principal investigators

Oncological institutions (in alphabetical order of cities)

Aachen: Universitätsklinikum, S. Wilop, M. Tometten; **Berlin:** Charité Universitätsmedizin, A. Korfel, U. Keller; Vivantes Klinikum Neukölln, M. de Wit; **Bielefeld:** Evangelisches Krankenhaus, F. Weissinger; **Bocholt:** St. Agnes Krankenhaus, U. Stark; **Bochum:** Augusta-Kranken-Anstalt, D. Behringer; Gemeinschaftspraxis für Hämatologie, Onkologie, Hämostaseologie und Palliativmedizin, U. Bückner, H. Nückel; Knappschaftskrankenhaus, R. Schroers; **Bottrop:** Knappschaftskrankenhaus, G. Trenn; **Bremen:** Klinikum Bremen Mitte, B. Hertenstein; **Darmstadt:** Klinikum, H. Bernhard; **Dortmund:** Klinikum, M. Heike, M.-A. Wörns; **Dresden:** Universitätsklinikum Carl Gustav Carus, F. Kroschinsky; Onkologische Gemeinschaftspraxis, G. Prange-Krex; **Duisburg:** HELIOS St. Johannes Klinik, A. Giagounidis, G.U. Grigoleit; Medizinisches Versorgungszentrum Onkologie, Standort Duisburg-Nord, J. Selbach; Sana Kliniken, S. Petrasch, J.S. Balleisen; **Düsseldorf:** Marien Hospital, H.-J. Schütte, A. Giagounidis; Universitätsklinikum, A. Dienst, U. Germing; **Essen:** Universitätsklinikum, U. Dührsen; Medizinisches Versorgungszentrum Hämatologie Onkologie, U. von Verschuer; Medizinisches Versorgungszentrum Essen-Nord, B. Schramm-Groß; Evangelisches Krankenhaus Werden, P. Reimer; **Fulda:** Klinikum, H.-G. Höffkes; **Goch:** Wilhelm-Anton-Hospital, V. Runde; **Halle/Saale:** Hämatologisch-onkologische Gemeinschaftspraxis, C. Spohn, R. Moeller; **Hamm:** St. Marien-Hospital, H. Dürk; **Hannover:** Medizinische Hochschule, D. Kofahl-Krause, M. Heuser; **Heidelberg:** Universitätsklinikum, M. Witzens-Harig, C. Müller-Tidow; **Heilbronn:** Klinikum am Gesundbrunnen, U.M. Martens; **Herne:** Marien Hospital, D. Strumberg; **Jena:** Universitätsklinikum, P. La Rosée, A. Hochhaus; **Kaiserslautern:** Westpfalz-Klinikum, H. Link, G. Held; **Kiel:** Universitätsklinikum Schleswig-Holstein, M. Kneba, C. Baldus; **Koblenz:** Stiftungsklinikum Mittelrhein, R. Naumann, J.M. Chemnitz; **Köln:** Krankenhaus Holweide, K. Schulte, C. Limmroth; **Leipzig:** Onkopraxis Probstheida, A. Schwarzer; Universitätsklinikum, D. Niederwieser, U. Platzbecker; **Lüdenscheid:** Klinikum, G. Heil, M. Schwalenberg; **Minden:** Johannes Wesling Klinikum, M. Grieshammer; **Mönchengladbach:** Medizinisches Versorgungszentrum Onkologie, C. Beck, M. Stephany; **Münster:** Universitätsklinikum, R. Mesters, I.E. Karsten; **Neumünster:** Friedrich-Ebert-Krankenhaus, H. Held, S. Mahlmann; **Oberhausen:** Praxis für Hämatologie und Onkologie, H. Steiniger; **Paderborn:** Brüderkrankenhaus St. Josef, T. Gaska; **Passau:** Klinikum, T. Südhoff; **Porta Westfalica:** Zentrum für Hämatologie und Onkologie Medizinisches Versorgungszentrum, C. Kreisel-Büstgens, E. Moorahrend; **Potsdam:** Klinikum Ernst von Bergmann, G. Maschmeyer, K. Jordan; **Recklinghausen:**

Knappschaftskrankenhaus, O. Kloke, M. Klein; Prosper-Hospital, T. Höhler; **Regensburg:** Universitätsklinikum, M. Grube, W. Herr; **Stuttgart:** Klinikum, D. Hahn; **Wuppertal:** HELIOS Universitätsklinikum, A. Raghavachar, O. Schmalz; Medizinisches Versorgungszentrum West, W. Fett; Petrus-Krankenhaus, M. Sandmann.

Nuclear medicine institutions (in alphabetical order of cities)

Aachen: Universitätsklinikum, T. Krohn; **Berlin:** Charité Universitätsmedizin, W. Brenner; Vivantes Klinikum Neukölln, M. Plotkin; **Bremen:** Zentrum für moderne Diagnostik (Zemodi), C. Franzius; **Dresden:** Universitätsklinikum Carl Gustav Carus, J. Kotzerke; **Düsseldorf/Jülich:** Universitätsklinikum, H. Hautzel; **Essen:** Universitätsklinikum, A. Bockisch; **Fulda:** Klinikum, A. Hertel; **Hannover:** Medizinische Hochschule, F. M. Bengel; **Heidelberg:** Universitätsklinikum, U. Haberkorn; **Jena:** Universitätsklinikum, M. Freesmeyer; **Kiel:** Universitätsklinikum Schleswig-Holstein, U. Lützen; **Koblenz:** Bundeswehrkrankenhaus, A. Klein; **Leipzig:** Universitätsklinikum, R. Kluge; **Lüdenscheid:** Klinikum, R. Larisch; **Minden:** Johannes Wesling Klinikum, E. Fricke, J. Holzinger; **Mönchengladbach:** Kliniken Maria Hilf, W. Schäfer; **Münster:** Universitätsklinikum, M. Weckesser; **Paderborn:** Brüderkrankenhaus St. Josef, F. Nyuyki; **Passau:** Klinikum, W. Römer; **Potsdam:** Klinikum Ernst von Bergmann, I. Brink; **Regensburg:** Universitätsklinikum, J. Marienhagen; **Stuttgart:** Klinikum, G. Pöpperl.

Medical biometry and biostatistics

Essen: Institut für Medizinische Informatik, Biometrie und Epidemiologie, Universität Duisburg-Essen, K.-H. Jöckel, M. Nonnemacher, J. Rekowski, A. Scherag, M. Neuhäuser.

Reference pathologists (in alphabetical order)

A.C. Feller, Universitätsklinikum Schleswig-Holstein, Campus Lübeck
M.L. Hansmann, Universitätsklinikum, Frankfurt/Main
W. Klapper, Universitätsklinikum Schleswig-Holstein, Campus Kiel (chairman)
P. Möller, Universitätsklinikum, Ulm
A. Rosenwald, Universitätsklinikum, Würzburg
H. Stein, Universitätsklinikum Benjamin Franklin, Berlin

Data and Safety Monitoring Board (in alphabetical order)

R. Andreesen, Universitätsklinikum, Regensburg (oncology)
F. Grünwald, Universitätsklinikum, Frankfurt/Main (nuclear medicine)
D. Hoelzer, Onkologikum, Frankfurt/Main (oncology, chairman)
W. Köpcke, Universitätsklinikum, Münster (biostatistics)
